# Metal‐Based Nanocatalysts via a Universal Design on Cellular Structure

**DOI:** 10.1002/advs.201902051

**Published:** 2019-11-26

**Authors:** Yajing Zhao, Xin Min, Zhengping Ding, Shuang Chen, Changzhi Ai, Zhenglian Liu, Tianzi Yang, Xiaowen Wu, Yan'gai Liu, Shiwei Lin, Zhaohui Huang, Peng Gao, Hui Wu, Minghao Fang

**Affiliations:** ^1^ Beijing Key Laboratory of Materials Utilization of Nonmetallic Minerals and Solid Wastes National Laboratory of Mineral Materials School of Materials Science and Technology China University of Geosciences (Beijing) Beijing 100083 P. R. China; ^2^ International Center for Quantum Materials and Electron Microscopy Laboratory School of Physics Peking University Beijing 100871 P. R. China; ^3^ State Key Laboratory of Marine Resource Utilization in South China Sea School of Materials Science and Engineering Hainan University Haikou 570228 P. R. China; ^4^ State Key Laboratory of New Ceramics and Fine Processing School of Materials Science and Engineering Tsinghua University Beijing 100084 China

**Keywords:** cellular structure, C‐N supports, hydrogen evolution reaction, metal‐based nanocatalysts, universal design

## Abstract

Metal‐based nanocatalysts supported on carbon have significant prospect for industry. However, a straightforward method for efficient and stable nanocatalysts still remains extremely challenging. Inspired by the structure and comptosition of cell walls and membranes, an ion chemical bond anchoring, an in situ carbonization coreduction process, is designed to obtain composite catalysts on N‐doped 2D carbon (C‐N) loaded with various noble and non‐noble metals (for example, Pt, Ru, Rh, Pd, Ag, Ir, Au, Co, and Ni) nanocatalysts. These 2 nm particles uniformly and stably bond with the C‐N support since the agglomeration and growth are suppressed by anchoring the metal ions on the cell wall and membrane during the carbonization and reduction reactions. The Pt@C‐N exhibits excellent catalytic activity and long‐term stability for the hydrogen evolution reaction, and the relative overpotential at 100 mA cm^−2^ is only 77 mV, which is much lower than that of commercial Pt/C and Pt single‐atom catalysts reported recently.

## Graphical abstract

A series of metal‐based nanocatalysts is prepared through a universal design based on various cellular structures. These uniform noble and non‐noble metal nanoparticles, such as Pt, Ru, Rh, Pd, Ag, Ir, Au, Co, and Ni, stably bond with a N‐doped carbon support, which leads to an excellent catalytic activity and long‐term stability for the hydrogen evolution reaction.

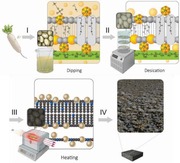

Metal‐based nanocatalysts have an irreplaceable role in energy conversion,^1^ chemical production,^2^ and automotive exhaust purification.^3^ Common metal nanocatalysts include noble metal^4^, ^5^, ^6^ or transition metal^7^ and alloys,^8^ which provide active sites for catalytic reactions. The substrate materials commonly used as supports for metal nanocrystals are oxides,^9^ sulfides,^10^ and carbon materials,^6^, ^11^ which mainly serve to stabilize the catalyst and strengthen charge transfer. Metal‐based nanocatalysts using carbon as supports (NM@C) own advantages of low‐cost, large specific surface area, good electrical conductivity, and high catalytic activity,^12^ which determine the practical application prospects.

There are many methods for directly preparing the NM@C catalysts with controlling particle size and enhancing stability,^13^ such as simple pyrolysis,^14^ wet chemical methods,^15^ and atomic layering deposition.^4^, ^16^ However, clear designs on the metal catalysts and carbon supports are still lacking, and their problems also limit the practical application of metal‐based nanocatalysts.^17^ Therefore, it is urgent to develop simple and effective methods to prepare efficient and stable NM@C catalysts.

Inspired by biological structures, a variety of materials utilizing the biological or biomimetic structures are developed. The biological cell wall and cell membrane are natural 2D structures with good ion permeability and exchangeability. Their basic structural units are cellulose, phospholipid bilayers, membrane proteins embedded in bilayers, and sugars and glycolipids bonded to the proteins. Many labile bonds, such as —C—OH, —C—O—, and —C—N—, are included in these structural units. According to these composition and structure, we designed a class of metal nanocatalysts supported on biocarbon by the ion exchange and chemical bond anchoring—in situ carbonization coreduction treatment. During the preparation process, the unstable bonds in the cell wall and the membrane are broken, and a more stable chemical bond is formed with inorganic metal ions (or clusters) in the solution; thus, the metal ions could be uniformly fixed on the surface of the cell wall and membrane. When the cell wall and membrane are in situ carbonized to form a 2D carbon material, the metal ions may also be simultaneously reduced to metal atoms to produce metal nanoparticles stably anchored on the surface of carbon. Additionally, in the above experimental design, since the proteins embedded in the cell membrane contain a large amount of N atoms, N may directly doped into the carbon during the carbonization process to improve the electronic state of the carbon support, resulting in excellent catalytic activity and stability superior to those of commercial Pt/C and other reported Pt single‐atom catalysts for the hydrogen evolution reaction (HER). Our method is simple, low‐cost, environmentally friendly, and universally applicable. It can be used to prepare various precious metal and nonprecious metal‐based nanocatalysts with nitrogenated carbon (C‐N) support. Furthermore, the resultant catalysts are competitive with other catalysts for fuel cell, electrocatalysis and energy chemical industry applications due to the outstanding advantages of their small and uniform particle size, strong bonding strength with the C‐N support, and abundant exposed active sites.

Using the above design principle as theoretical support, we selected white radish as the representative plant and chloroplatinic acid (H_2_PtCl_6_·6H_2_O) as the precursor solution and then carried out the experiment as follows (**Figure** **1**a). First, the white radish was sliced and immersed in H_2_PtCl_6_·6H_2_O solution for minutes so that the chloroplatinate ions could fully permeate the cell structure through free diffusion, bond, and stably combine with the cell wall and membrane. The impregnated white radish slices were rapidly frozen and further dried by freeze‐drying to ensure the stability of the multilayered 2D structure and inhibit agglomeration or crystallization of the intracellular chloroplatinic acid (In an ordinary normal drying process, crystallization will occur once the concentration of the solution exceeds the solubility.). Finally, the dried slices were calcined in an Ar‐protected tube furnace to realize in situ carbonization of cell wall and membrane, nitrogen doping, and in situ reduction and anchoring of platinum ions (Figure 1b). Finally, our catalyst was obtained by Pt nanoparticles dispersed within a nitrogenated 2D carbon support (Pt@C‐N).

**Figure 1:**
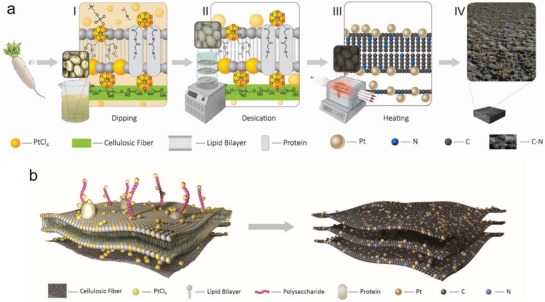
Schematic diagram of the synthesis process of the Pt@C‐N catalyst. a) Schematic illustration of the synthesis process. Inset: chemical bonding between [PtCl_6_]^2−^ and the cell wall and membrane after the dipping (I) and desiccation (II) processes, and a schematic illustration for the atomic structure (III) and structural features (IV) of Pt@C‐N sample after the in situ carbon coreduction process. b) The state of the [PtCl_6_]^2−^ cluster bonded to the cell wall and membrane (left) after desiccation and Pt nanoparticles anchored on the nitrogenated 2D carbon (right).

In the above process, the sliced white radish is initially white and then golden yellow after being immersed in the chloroplatinic acid solution and freeze‐dried (Figure S1, Supporting Information), and the final product Pt@C‐N obtained by in situ carbonization coreduction is black (Figure S2, Supporting Information). After heating in argon at 600 °C, Pt@C‐N still maintains a porous structure composed of 2D carbon flakes (inset of **Figure** **2**a, which is similar to the porous cell structure of white radish), and the Pt nanoparticles are uniformly distributed on C‐N support (Figure 2a). In the X‐ray diffraction (XRD) results (Figure 2b), the broad diffraction peak at 23° is from C‐N with low graphitization (Figure S3, Supporting Information), while the sharp diffraction peaks are derived from cubic Pt (powder diffraction file (PDF) No. 87‐646). These particles are in nanosize and encapsulated in C‐N, so their diffraction peaks are also broadened. X‐ray photoelectron spectroscopy (XPS) analysis further proved that Pt, C, N, and O are present in Pt@C‐N (Figures S4 and S5, Supporting Information). Pt and C have a certain chemical bond (Figure 2c), C and N form a bond like C_2_N (Figure 2d,e), and O is mainly from air and water adsorbed on the sample. These chemically bonding will result in a stronger force between Pt and C‐N support. The corresponding theoretical simulation calculations (Figure S6, Supporting Information) further confirm that when Pt is combined with C‐N, the structure is more stable than that obtained from direct combination with the carbon material. Thus, the Pt@C‐N composite could have better structural and performance stability in catalytic reactions.

**Figure 2:**
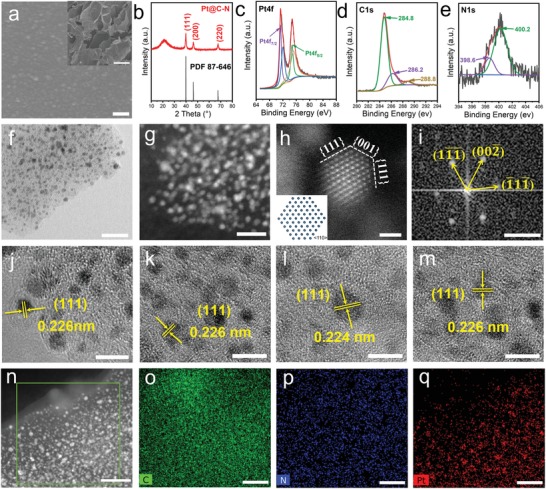
The morphology and structure of Pt@C‐N catalysts. a) SEM image of Pt@C‐N; scale bar: 100 nm. Inset: morphology of Pt@C‐N at lower magnification; scale bar: 100 µm. b) XRD pattern of Pt@C‐N. c–e) XPS spectra of Pt4f (c), C1s (d), and N1s (e) in Pt@C‐N. f) TEM image of Pt@C‐N; scale bar: 20 nm. g) AC‐STEM image of Pt@C‐N; scale bar: 10 nm. h) Atomic‐resolution HADDF‐STEM images of Pt@C‐N and i) corresponding FFT pattern; scale bar: h) 1 nm and i) 5 nm^−1^. Inset: corresponding atomic model. j–m) HRTEM images of Pt@C‐N catalysts prepared through different biological matrix materials: j) white radish, k) dry nostoc flagelliforme, l) seaweed, and m) potato; scale bar: 5 nm. n–q) STEM image and STEM‐EDS element mapping of Pt@C‐N; scale bars: n) 30 nm and o–q) 20 nm.

In the transmission electron microscopy (TEM) (Figure 2f) and scanning transmission electron microscopy (STEM) (Figure 2g) images, the Pt@C‐N structure with a unique morphology can be clearly seen. The Pt nanoparticles have a diameter of ≈2 ± 0.5 nm and are uniformly dispersed on the surface of C‐N. The atomic structure of the Pt nanoparticles was further analyzed by atomic resolution spherical aberration corrected high angle annular dark field (HAADF) STEM (Figure 2h) and its fast Fourier transformation (FFT) image (Figure 2i). The distances of the crystal lattice after Fourier transform are 0.225 nm (4.445 nm^−1^) and 0.195 nm (5.134 nm^−1^), which correspond to the {111} and {002} planes, respectively, of face‐centered cubic Pt along the <110> ribbon axis. Moreover, the corresponding atomic model (inset of Figure 2h) indicates that the {111} crystal plane in the direction of [110] is fully exposed. The STEM‐coupled energy‐dispersive X‐ray spectroscopy (STEM‐EDS) elemental mapping (Figure 2n–r) also confirms that the Pt nanoparticles are uniformly distributed on the surface of C‐N. The TEM image of Pt@C‐N after ball milling for 24 h and sonication for 2 h also proved its excellent stability (Figure S7, Supporting Information). The Pt content in the Pt@C‐N sample was calculated to be ≈22.8% from the inductively coupled plasma–optical emission spectroscopy (ICP‐OES). The high content of Pt nanoparticles will provide more active sites for electrochemical reactions.

We carefully studied the Pt@C‐N samples synthesized with different concentrations of impregnation solution and different carbonization coreduction temperatures, and they also have a porous microscopic morphology with 2D carbon flakes (Figures S8 and S9, Supporting Information). Pt nanoparticles are stably supported on C‐N to obtain the special Pt@C‐N structure (Figures S10–S15, Supporting Information). When the concentration of the solution increases from 0.04 to 0.12 m, the size of Pt nanoparticles increases mildly from 1.5 to 3.5 nm. But the particle size was influenced by the heat‐treatment temperature, i.e., the particle size increases from ≈1.8 to 5 nm when the temperature is raised from 400 to 800 °C. All these Pt nanoparticles can be categorized as two types according to their particle size (Figures S14 and S15, Supporting Information): Pt particles with a particle size of ≈2 nm (These nanoparticles could be found in all samples prepared at different concentrations or different temperatures.) and Pt particles with an uncertain size.

Based on the above results, we analyzed the synthesis mechanism of the Pt@C‐N samples combined with the structure and composition of plant cells. When the white radish slices are impregnated in the chloroplatinic acid solution, [PtCl_6_]^2−^ ion diffuses through the multilayer cell wall and membrane and penetrates the slices. Meanwhile, ion exchange occurs between [PtCl_6_]^2−^ ion and the cell wall or membrane, resulting in the following three scenarios. First, the [PtCl_6_]^2−^ ion may replace the hydroxyl groups on the cellulose to form the special structure —C—[PtCl_6_]—C— in cell wall (Equation (S1) in the Supporting Information; a similar structure may also be formed on the polysaccharide chain in cell membrane); second, the ion may replace the polar phospholipid head of the phospholipid molecule in the cell membrane to form the structure —C—[PtCl_6_]—C— with its hydrophobic nonpolar tail (Equation (S2), Supporting Information); and third, the ion may replace the alanine in membrane protein and form the structure —C(=O)—[PtCl_6_]—(O=)C— with the amino acid (Equation (S3), Supporting Information). After the impregnation process, most [PtCl_6_]^2−^ ions can be anchored on the surface of the cell wall and membrane through these stable chemical bonds. However, others are still freely dispersed in solution in the cells or between them, failing to bond with the wall and membrane (Figure 1a‐I). Freeze drying is utilized to maintain the porous structure of natural plants and to inhibit agglomeration and gravity sedimentation of freely dispersed [PtCl_6_]^2−^ ions. After the desiccation process, these chloroplatinate are uniformly deposited on the surface of the cell wall and membrane. Thus, two different states of the [PtCl_6_]^2−^ are obtained: chemically bonding and ordinary accumulating on the surface of the cell wall and membrane (Figure 1a‐II,b‐I). During the in situ carbonization coreduction process, all the chloroplatinate ions are reduced to form Pt nanoparticles, and the N in the membrane protein also directly participates in the carbonization process of the cell wall and membrane interacting with C to form a 2D C‐N structure. The chloroplatinate ions anchored on the cell structure are directly reduced to Pt atoms, then Pt nanoparticles are formed due to the migration of adjacent Pt atoms, which further result a stable chemical bond between the Pt nanoparticles and C‐N. While the accumulated chloroplatinate ions on cell wall and membrane are directly reduced to form Pt nanoparticles, which are only adsorbed on the surface of C‐N. Finally, the Pt@C‐N material is obtained (Figure 1a‐III–IV,b‐II).

When the heating temperature increases, the size of most Pt nanoparticles does not increase significantly due to their stable chemical bond between the chloroplatinate ions and the cell structure. However, the accumulated chloroplatinate ions on the cell structure are more easily to agglomerate at high temperature due to the lack of bonding interaction; thus, the size of some Pt nanoparticles increases with the increasing temperature. For another, when the concentration of solution increases, the amount of chloroplatinate ions anchored on the cell structure remains almost constant because there is no significant change in surface area of the cell wall and membrane for similar slices. However, the size of the chloroplatinate clusters on the cell wall and membrane will increase as the solution concentration increases, but due to inhibition from the freeze‐drying process, the changes in particle size before and after heat treatment is still small. Thus, the Pt nanoparticles could keep almost unchanged with the increasing concentration. Therefore, based on any plant and its cell structure, we believe that stable 2D C‐N structure anchored with diverse size‐controlled metal nanoparticles can be achieved through our simple design described above, as long as the parameters such as the concentration of solution and the reaction temperature are controlled.

To verify the feasibility of our theoretical design, a variety of plants and different precious metals or transition metal salt solutions were selected for experiments. Pt@C‐N materials with similar structures and morphologies were obtained based on different plants, such as white radish, dry nostoc flagelliforme, seaweed and potato (Figures S16 and S17, Supporting Information). The diameter of all the Pt nanoparticles is ≈2 ± 0.7 nm based on the high resolution TEM (HRTEM) images (Figure 2j–m). The crystal plane fringe is ≈0.226 nm, indicating the fully exposed {111} crystal plane. The carbon matrix also consists of C and N to form C‐N like the C_2_N structure, and Pt nanoparticles bond with C‐N and obtain structurally stable Pt@C‐N composite catalysts (Figures S18, Supporting Information). Various metal‐based nanocatalysts loaded on C‐N support such as Co@C‐N, Ni@C‐N, Ru@C‐N, Rh@C‐N, Pd@C‐N, Ag@C‐N, Ir@C‐N, and Au@C‐N were also prepared separately according to our design (**Figure** **3**, Figures S19–S21, Supporting Information). The particle sizes of the different nanometals are slightly different (≈1–5 nm), which may be related to the difference in bond strength between the ions and cell structure, or in migration ability of metal atoms during heat treatment. If the designed process is further optimized, the particle size can be more uniform. These extended results fully confirm that the designed “complete metal salt solution impregnation—freeze‐drying—in situ carbonization coreduction” process can be used to successfully prepare a variety of precious‐metal‐ and nonprecious‐metal‐based C‐N nanocatalysts, which is simple and can be universally applied, and the product has a controllable particle size and good structural stability.

**Figure 3:**
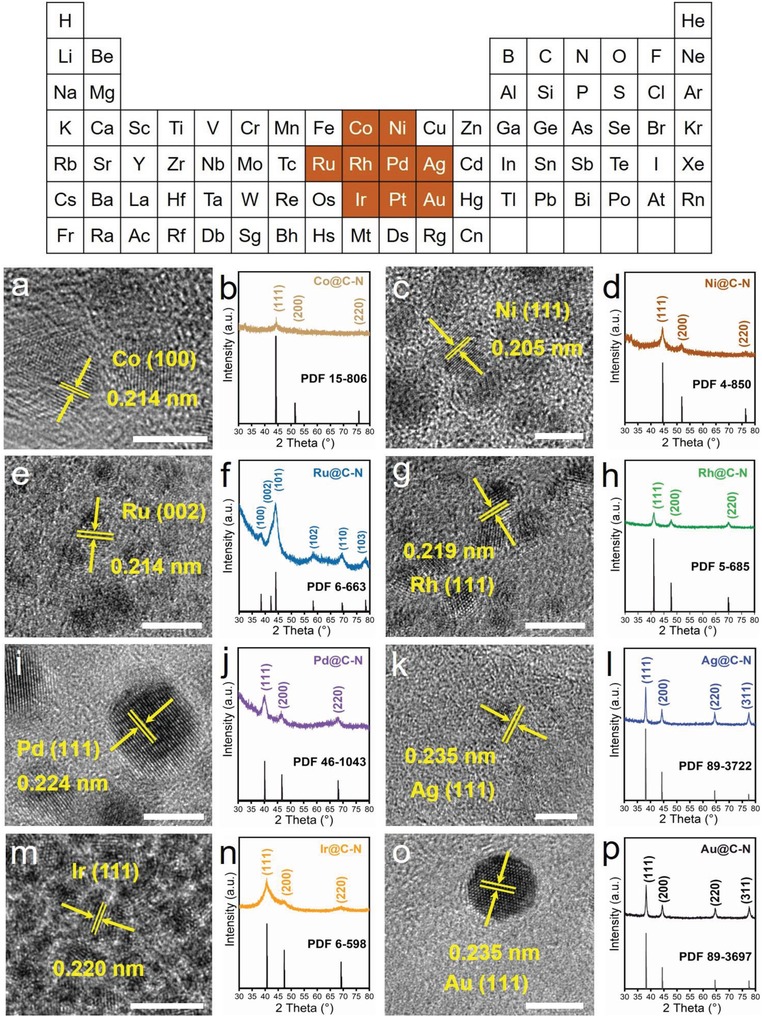
Morphology and structure of various metal‐based catalysts on C‐N support. a–p) HRTEM images of Co@C‐N (a), Ni@C‐N (c), Ru@C‐N (e), Rh@C‐N (g), Pd@C‐N (i), Ag@C‐N (k), Ir@C‐N (m), and Au@C‐N (o) and XRD patterns of Co@C‐N (b), Ni@C‐N (d), Ru@C‐N (f), Rh@C‐N (h), Pd@C‐N (j), Ag@C‐N (l), Ir@C‐N (n), and Au@C‐N (p).

We studied the HER performance of the various metal‐based C‐N nanocatalysts in 0.5 m H_2_SO_4_ solution. Commercial Pt/C and as‐prepared C‐N are also tested for comparison under the same conditions. The Pt@C‐N catalyst prepared by heating at 600 °C after complete impregnation in a 0.08 m chloroplatinic acid solution has the best catalytic activity (Figures S22–S26 and Table S1, Supporting Information), and the relative overpotential at a current density of 100 mA cm^−2^ is just 77 mV versus the reversible hydrogen electrode (RHE), which is significantly lower than the relative overpotential (100 mV) of commercial Pt/C and samples produced under other conditions (**Figure** **4**a). At the same time, this catalyst also showed a small initial overpotential closed to 0, which is only slightly higher than that of the commercial Pt/C. More importantly, the Tafel slope of Pt@C‐N is calculated to be 23 mV dec^−1^, which is 9 mV dec^−1^ lower than the Tafel slope of commercial Pt/C (32 mV dec^−1^) (Figure 4b), indicating that the HER mechanism is a Volmer–Tafel process. Further detailed analysis reveals that the HER reaction is a hydrogen ion diffusion rate‐controlled process (Figure S27, Supporting Information). Under this condition, the initial overpotential, Tafel slope and relative overpotential (100 mA cm^−2^) of Pt@C‐N (using white radish as the plant matrix) are also much lower than those of Pt@C‐N prepared in different plants (Figure 4d, Figure S28, Supporting Information) or other metal‐based nanocatalysts loaded on C‐N (Figures S29 and S30, Supporting Information). The possible reason for this difference is that the preparation process and structure, and the catalytic selectivity of these nanocatalysts have not been optimized in detail. Compared with the HER catalysts reported recently (including metal single‐atom catalysts, metal or alloy nanocatalysts, and catalysts such as metal oxides, sulfides, and phosphides), the Tafel slope and relative overpotential (10 mA cm^−2^) of the Pt@C‐N reported herein are also much lower (Figure 4f,g, Figures S31 and S32, Table S2, Supporting Information). Such a low Tafel slope means that the hydrogen evolution rate of Pt@C‐N will increase rapidly with the increase of overpotential, which makes it a potential catalyst for practical application. The electrochemical stability of Pt@C‐N and Pt/C was analyzed by a long‐term operation at a fixed overpotential of 30 mV (Figure 4e). After 10 000 s, the attenuation of Pt@C‐N (15%) was significantly less than that of commercial Pt/C (42%), but slightly higher than the Pt single‐atom catalysts.^11^ The nearly invariable structure of Pt@C‐N after long‐term electrochemical testing also clearly indicates that Pt@C‐N has a good electrochemical stability for long‐term electrochemical reactions when compared with that of the commercial Pt/C (Figures S33 and S34, Supporting Information), which can be attributed to the strong chemical bonding between Pt nanoparticles and the C‐N matrix.

**Figure 4:**
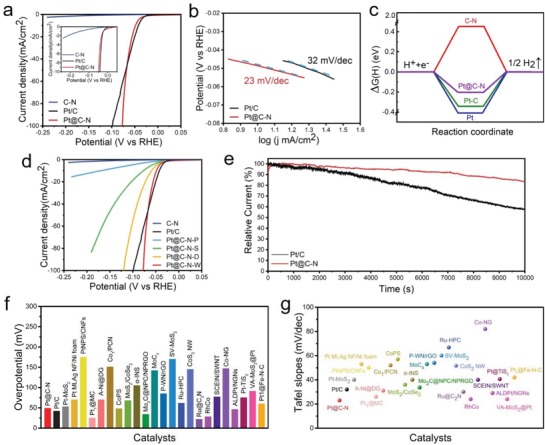
HER activity of the electrocatalysts in 0.5 m H_2_SO_4_. a) Polarization curves of C‐N, Pt@C‐N, and commercial Pt/C at a scan rate of 10 mV s^−1^. Inset: local enlarged polarization curves of C‐N, Pt@C‐N, and commercial Pt/C. b) Tafel slopes obtained from the polarization curves in (a). c) Reaction mechanism for the HER, calculating Δ*G*_H*_ for atomic H adsorption on C‐N, Pt, Pt/C, and Pt@C‐N. d) Polarization curves of electrocatalysts based on various matrix materials. Pt@C‐N‐P, Pt@C‐N‐S, Pt@C‐N‐D, and Pt@C‐N‐W represent the Pt@C‐N catalysts based on the potato, seaweed, dry nostoc flagelliforme, and white radish, respectively. e) Long‐term running of Pt@C‐N and commercial Pt/C electrocatalysts at the same overpotential of 30 mV. f,g) Comparison of overpotential at 10 mA cm^−2^ (f) and Tafel slopes (g) of Pt@C‐N, commercial Pt/C, and recently reported HER catalysts in 0.5 m H_2_SO_4_.

Combined with the first‐principles density functional theory (DFT) calculation,^18^ we discuss the excellent electrocatalytic activity of Pt@C‐N in the HER process. First, the doping of N atoms into the 2D carbon support can improve the electronic state on the surface of carbon, providing more paths for charge transfer and further improving the catalytic activity of the surface‐loaded metal nanocatalysts (Figure S35, Supporting Information). Second, in the optimized Pt@C‐N material, the two nm Pt nanoparticles are uniformly anchored on C‐N support with a large specific surface area. The catalytic active sites on the {111} crystal plane of the Pt nanoparticles are fully exposed, thereby maximizing the use of Pt in HER. In addition, Pt@C‐N in this paper is obtained by heat treatment at a high temperature of 600 °C, and most of the Pt nanoparticles are anchored by a strong chemical bond to the support materials. The stability of the metal‐based nanocatalysts is excellent, which can effectively suppress abnormal growth, agglomeration and shedding of Pt nanoparticles during the electrochemical reaction, resulting in the good stability of the HER performance. Finally, the Gibbs free energy (Δ*G*_H*_) of the hydrogen atoms adsorbed on the surface of catalysts such as C‐N, Pt, Pt/C, and Pt@C‐N was further calculated by DFT to understand the high electrocatalytic activity in the HER process (Figures S36–S39, Supporting Information). When H atoms are adsorbed on the exposed {111} crystal plane of Pt_13_, it is easier to simultaneously bond with three atoms at the same crystal plane, and the adsorption binding energy is only 0.03 eV, which is much lower than that in other cases. The Δ*G*_H*_ of Pt@C‐N catalyst (−0.23 eV) also exhibits a smaller negative Gibbs free energy than others (Figure 4c). The C‐N support can significantly improve the catalytic activity of the Pt nanoparticles, so Pt@C‐N has an excellent electrocatalytic HER performance.

In summary, we designed a universal route to successfully support Pt nanocatalysts on C‐N by the designed “complete metal salt solution impregnation—freeze‐drying—in situ carbonization coreduction” process. Compared with commercial Pt/C, our elaborate Pt@C‐N catalyst has a remarkable HER activity and electrochemical stability, originating from the small and uniform particle size, and the strong chemical bonds with the C‐N matrix. The facile, low‐cost, universality, and wide applicability of this method can be further used to prepare various precious metal or nonprecious metal nanoparticles on C‐N support using various biomaterials as the base system. The method opens a new approach to produce efficient and stable supported metal‐based nanocatalysts.

## Experimental Section

Details of all the materials, experiments, characterizations, electrocatalytic measurements, and DFT calculation used in this experiment are provided in the Supporting Information.

## Conflict of Interest

The authors declare no conflict of interest.

## Supporting information

Supporting Information
