# Supplementary material for: Metal‐Based Nanocatalysts via a Universal Design on Cellular Structure
Source: Adv Sci (Weinh). 2019 Nov 26;7(3):1902051. doi: 10.1002/advs.201902051 (PMC7001642; doi:10.1002/advs.201902051)
Supplement: Supplementary file 1 — Supporting Information [file ADVS-7-1902051-s001.pdf]

Copyright WILEY-VCH Verlag GmbH & Co. KGaA, 69469 Weinheim, Germany, 2019.

ADVANCED SCIENCE
Open Access

Supporting Information

for *Adv. Sci.,* DOI: 10.1002/advs.201902051

Metal-Based Nanocatalysts via a Universal Design on Cellular Structure

Yajing Zhao, Xin Min, Zhengping Ding, Shuang Chen, Changzhi Ai, Zhenglian Liu, Tianzi Yang, Xiaowen Wu, Yan'gai Liu, Shiwei Lin,* Zhaohui Huang, Peng Gao,* Hui Wu,* and Minghao Fang**

Supporting Information

Metal-Based Nanocatalysts via a Universal Design on Cellular Structure

Yajing Zhao[1†], Xin Min[1†], Zhengping Ding[2†], Shuang Chen[1], Changzhi Ai[3], Zhenglian Liu[1], Tianzi Yang[1], Xiaowen Wu[1], Yan'gai Liu[1], Shiwei Lin[3]*, Zhaohui Huang[1], Peng Gao[2]*, Hui Wu[4]*, Minghao Fang[1]**

The PDF file includes:

Section S1. Materials and Experimental

Section S1.1 Materials

Section S1.2 Experimental

Section S1.3 Characterizations

Section S1.4 Electrocatalytic measurements

Section S1.5 DFT calculation

Section S2. Supplementary Notes

Section S2.1 XPS analysis of the Pt@C-N.

Section S2.2 The reaction is a hydrogen atom diffusion rate-controlled process.

Fig. S1. Photograph of as-prepared Pt@WRC samples during the impregnated and drying progress.

Fig. S2 Photograph of as-prepared Pt@WRC catalyst on filter paper.

Fig. S3. Raman spectrum of the electrocatalysts of Pt@WRC.

Fig. S4. The whole spectrum of the XRS of Pt@WRC.

Fig. S5. XPS data of P (a), S (b), and K (c) in the Pt@C-N sample.

Fig. S6. The schematic diagram of the stability bonds between Pt_{13} and C and C/N.

Fig. S7. TEM image of the Pt@C-N catalyst after ball milling for 24 h and sonication for 2 h.

Fig. S8. SEM images of the Pt@WRC catalysts calcined at different temperature.

Fig. S9. SEM images of the Pt@WRC catalysts impregnated in different concentration of H_2PtCl_6 aqueous solution.

Fig. S10. XRD patterns of the electrocatalysts under different concentration of H_2PtCl_6 aqueous solution.

Fig. S11. XRD patterns of the electrocatalysts at different calcined temperature.

Fig. S12. XPS spectrum of the electrocatalysts calcined at different temperature.

Fig. S13. XPS spectrum of the electrocatalysts calcined at impregnated in different concentration of H_2PtCl_6 aqueous solution.

Fig. S14. HRTEM image of Pt@C-N electrocatalyst calcined at different temperature.

Fig. S15. HRTEM image of Pt@C-N electrocatalyst calcined at 600 °C and impregnated in different concentration of H_2PtCl_6 aqueous solution.

Fig. S16. TEM images of the electrocatalysts on different supported materials.

Fig. S17. XRD patterns of the electrocatalysts on different supported materials.

Fig. S18. XPS spectrum of the electrocatalysts on different supported materials.

Fig. S19. TEM images of different electrocatalysts.

Fig. S20. TEM images of different electrocatalysts.

Fig. S21. STEM-EDS images of the Ru@C-N electrocatalysts, Scale bars, 3 nm.

Fig. S22. Raman spectra of the Pt@C-N-W electrocatalysts of calcined at different temperature.

Fig. S23. Polarization curves of Pt@C-N in different concentration of H_2PtCl_6 solution.

Fig. S24. Polarization curves of Pt@C-N calcined at different temperature.

Fig. S25. Tafel slop of Pt@C-N electrocatalysts.

Fig. S26. HER activity of different Pt@C-N samples with same Pt mass loading.

Fig. S27. CV curves of Pt@C-N catalyst at different scanning rate and log(i) versus log(v) of the as-prepared sample.

Fig. S28. Tafel slopes curves of electrocatalysts on various matrix materials.

Fig. S29. Polarization curves of diverse metal-based electrocatalysts.

Fig. S30. Tafel slopes curves of diverse metal-based electrocatalysts.

Fig. S31. Local enlarged polarization curves of electrocatalyst on various matrix.

Fig. S32. Local enlarged polarization curves of metal-based electrocatalysts.

Fig. S33. TEM images of Pt@C-N before and after stability test.

Fig. S34. TEM images of commercial Pt/C before and after stability test.

Fig. S35. Charge density difference maps for H adsorption onto the right above of the Pt_{13} (111) plate.

Fig. S36. The schematic diagram of the H atom absorbed at different sites of Pt_{13} (111).

Fig. S37. Structures of H atom adsorbed on the C/N materials.

Fig. S38. Structures of H atom adsorbed on the pure Pt_{13}.

Fig. S39. Structures of H atom adsorbed on the C of Pt@C-N catalyst.

Eq. S1-S4

Table S1: The $R_{ID/IG}$ values calculated from the Raman spectra of the Pt@C-N-W calcined at different temperature.

Table S2: Summary of some recently reported representative HER electrocatalysts in 0.5 M H_2SO_4 electrolytes.

Section S1. Materials and Experimental

Section S1.1 Materials

The biological materials (white radish (WRC), potato (PTC), seaweed (SWC) and dry nostoc flagelliforme (DFC)), applied as matrix materials, are purchased in the local market. The noble metals used in this experiment are chloroplatinic acid ($H_2PtCl_6 \cdot 6H_2O$), chloroauric acid ($HAuCl_4 \cdot 4H_2O$), rhodium chloride ($RhCl_3 \cdot 3H_2O$), ruthenium chloride ($RuCl_3$), palladium chloride ($PdCl_2$) and iridium chloride hydrate ($IrCl_3 \cdot xH_2O$), which are bought in Aladdin's official website. While the no-noble metals are bought from Beijing Chemical Reagent Ltd, such as cobalt-nitrate hexahydrate ($Co(NO_3)_4 \cdot 6H_2O$) and nickel nitrate hexahydrate ($Ni(NO_3)_2 \cdot 6H_2O$). Beyond that the others chemical reagents are analytically pure and bought from Beijing Chemical Reagent Ltd.

Section S1.2 Experimental

The biological materials WRC, PTC, SWC, and DFC are chosen as the source of porous biological materials. Exampled by WRC, WRC are preprocessed in the deionized water firstly. Then, the pre-prepared WRC are impregnated in the H_2PtCl_6 aqueous solution with different concentration (such as 0.04 mol/L, 0.06 mol/L, 0.08 mol/L, 0.10 mol/L and 0.12 mol/L) in brown reagent bottle. To maintain the morphological integrity of the biological materials, the impregnated WRC are further transferred to the vacuum freeze dryer for 72 h at -60 °C, and then the Pt@WRC catalyst precursor is obtained. Furthermore, the precursor is annealed at different temperature (400 °C, 500 °C, 600 °C, 700 °C and 800 °C) at Ar atmosphere for 2 h with the rate of 2 °C/min. The Pt@C-N samples are obtained and further optimized according to the HER activity. Different metal-based nanoparticles (Co, Ni, Ru, Rh, Pd, Ag, Ir and Au) are also explored under the optimized conditions.

Section S1.3 Characterizations

The micro-morphology of all the electrocatalysts are measured by SEM (Zeiss Supra-55). For transmission electron microscopy characterization, the samples were

dispersed onto a copper grid coated with a thin holey carbon film. High-resolution transmission electron microscopy of all catalysts was carried out on a FEI Tecnai G2 F30 microscope. High-angle annular dark field (HAADF) STEM images were acquired from an aberration-corrected FEI Titan Cubed Themis G2 microscope operated at 300 kV equipped with an X-FEG gun and Bruker Super-X EDX detectors. During image acquisition, the beam current was 45~100 pA, the convergence semi-angle was 30 mrad, and a collection semi-angle snap was in the range of 80-379 mrad. The STEM-EDX mapping was collected with a beam current of 150 pA and counts ranging from 1k to 5k cps for about 5 min. XRD studies are on a High-Power X-Ray Diffractometer (Bruker, D8 Advance) at a scanning rate of 8°/min. The Raman spectroscopy (Model PerkinElmer 400 + RamanMicro300) is tested with a light source of 532 nm and the range of the tested wavenumber is 200 nm-800 nm. X-ray photoelectron spectroscopy measurements are performed using an X-ray photoelectron spectrometer (Escalab 250Xi). The Pt content in the Pt@C-N electrocatalyst is measured by ICP-OES (PerkinElmer 8300).

Section S1.4 Electrocatalytic measurements

The catalyst dispersion or ink was prepared by mixing 5 mg catalyst in a 1 ml mixture of 750 μl ethyl alcohol, 200 μl ultrapure water and 50 μl 5 wt% Nafion solution. Then, the mixture solution was ultrasonicated for 30 min. For the Pt@C-N catalyst, after dispersing 5 mg catalyst in the 1 ml mixture, the concentration of Pt in the catalyst was 1.14 mg/ml. Ten microliters of the ink was dropped on freshly polished glassy carbon electrodes (diameter= 5 mm) to equally load the electrodes. The loading of Pt on the electrodes was 0.58 mg/cm^2 (58 μg/cm^2). The commercial Pt/C is purchased from the Shanghai Heson Electric co. LTD for comparison. The loading of Pt in the Pt/C is about 20 wt%, and the amount of Pt on electrodes are 51 μg/cm^2 for commercial Pt/C. The similar amounts of Pt in the cases of Pt@C-N and Pt/C confirm that the comparison between commercial Pt/C and Pt@C-N are effective herein.

All electrochemical tests, including linear sweep voltammetry (LSV) and cyclic voltammetry (CV), were measured by a conventional three-electrode electrochemical

configuration in 0.5 M H_2SO_4 solution using CHE660. An L-form glassy carbon electrode was used as the working electrode, while a carbon rod electrode and Ag/AgCl (with saturated KCl) were used as the counter and reference electrodes, respectively. LSV was carried out with a scan rate of 10 mV/s in a potential window from -0.08 mV to 0.04 mV, meanwhile the stability measurements (i-t) were conducted at the overpotential of 30 mV. After the stability test, the glass carbon electrode was sonicated for 5 min in ethanol, then put into an oven at 80 ℃ for minutes and prepared for the TEM measurement. All potentials measured were calibrated to the reversible hydrogen electrode (RHE) using the following equation:

$$E_{RHE} = E_{Ag/AgCl} + 0.197 + 0.059 \times pH$$

The surface of the working electrode was bubbled with H_2 during the HER. To accelerate the diffusion of H_2, all the LSV polarization curves for the HER were IR-corrected with 80% IR compensation. The correction was calculated by the following equation:

$$E_{(corrected)} = E_{(measured)} - iR_s$$

Section S1.5 DFT calculation

Based on DFT, the Vienna ab initio simulation package (VASP) was used in all calculations[1-3]. The exchange-correlation interactions using the generalized gradient approximation (GGA) of the Perdew-Burke-Ernzerhof functional (PBE type) were applied[4, 5]. The plane-wave cut-off energy was set to 400 eV, and the Brillouin-zone integration was set to a k-mesh of 3×3×3. The energy convergence criterium for atomic structures was 10^{-4} eV/atom, and the convergence threshold for the forces was 0.01 eV/Å. To avoid interactions between neighbouring slices due to lattice periodicity, a vacuum slab of 15 Å was introduced in the z direction. Furthermore, the activity of hydrogen evolution was described by the Gibb's free energy difference. The Gibb's free energy difference (ΔG_H) was calculated by the equation: $\Delta G_H = E_{(adsorbent+H)} - E_{(adsorbent)} - 1/2E_{(H_2)} + \Delta E_{ZPE} - T\Delta S$, where $E_{(adsorbent+H)}$, $E_{(adsorbent)}$ and $E_{(H_2)}$ are the total energy of the adsorbent and the adsorbed H atom, the energy of the adsorbent and the energy of a H_2 molecule[6], respectively, ΔE_{ZPE} is the zero-point

energy difference, and TΔS is considered to be approximately 0.2 eV at room temperature.

References

[1] G. Kresse and D. Joubert. *Phys. Rev. B* **1999**, *59*, 3.

[2] G. Kresse and J. Furthmüller. *Phys. Rev. B* **1996**, *54*, 16.

[3] P. E. Blöchl. *Phys. Rev. B* **1994** *50*, 24.

[4] J. P. Perdew, K. Burke and M. Ernzerhof. *Phys. Rev. Lett.* **1996** *77*, 18.

[5] Y. Zhang and W. Yang. *Phys. Rev. Lett.* **1998** *80*, 4.

[6] Z. Chen, Y. Song, J. Cai, X. Zheng, D. Han, Y. Wu, Y. Zang, S. Niu, Y. Liu, J. Zhu, X. Liu and G. Wang. *Angew Chem. Int. Ed. Engl.* **2018** *57*, 18.

Section S2. Supplementary Notes

Section S2.1. XPS analysis of the Pt@C-N.

Three special peaks are occurred in C1s, such as C-C bind at 284.8 eV, C-N bind at 286.2 eV and C-heteroatom at 288.8 eV. Meanwhile, two peaks of C-NH_2 and C-N pyrazine-like binds are at 398.6eV and 400.2 eV respectively in N 1s. In the O 1s binding energy, the positions at 530.9 eV, 532.1 eV and 533.6 eV show the characteristic peaks of O_2, C=O and H-O-H respectively. Noting that the C 1s and N 1s in the biological materials are similar with C_2N materials, thus the biological materials could be regarded as a C_2N-like materials. Besides, in the XPS spectrum of Pt, Pt^0 attributes to the unique peaks of Pt $4f_{5/2}$ and Pt $4f_{7/2}$ at 74.7 eV and 71.3 eV binding energy while the two peaks of 72.0 eV and 75.2 eV in the spectrum origin from Pt^{2+}. However, there is no extra peaks in XRD pattern expect Pt, indicating no PtO or $Pt(OH)_2$ exists in Pt@C-N catalyst. Thereby, Pt^{2+} maybe origins from a certain chemical bond between Pt and C or the oxidation during the measurement. Moreover, the microelements P, S, and K in the Pt@C-N sample were also investigated by XPS, as shown in Fig. S5. The intensities of the XPS peaks of P and S are very weak, which indicated that their concentrations are very low. The peak of P is located at 133.6 eV, coinciding with the binding energy of P-O bond in H_3PO4.[1] This special P-O bond might be resulted from the strong phosphate group, originating from the phospholipid bilayer in the cell membrane. No peaks for P-C bond or O-P-C bond or Pt-P bond can be observed in this data. For the S element, two peaks at 163.1 eV and 168.8 eV can be found in the XPS spectrum, which agrees well with the binding energy of S-S and S-O bond in terminal S polysulfide and sulfate, respectively.[2] Similarly, no S-C bond or O-S-C bond or Pt-S bond could be found in the result. However, the element K can hardly be detected in the XPS data, which might be dissolved out during the previously impregnation treatment. It could be concluded that the tiny amounts of elements P, S, and K do not bond with both the Pt catalysts and C-N supports, which may have negligible influences on the catalytic performances. Thus, these microelements are overlooked in the following studies.

Section S2.2. The reaction is a hydrogen atom diffusion rate-controlled process.

In order to explore the reaction kinetics of the as-prepared Pt@C-N, the CV measurements of the Pt@C-N at different scan rates were recorded and are shown in Fig. S29. It is well-known that the relation between the current (I) and the scanning rate (v) can be described by the following equations:

$$\mathrm{i} = \mathrm{a}v^{b}$$

$$\log(i) = blog(v) + \log(a)$$

where a and b are both constants. The value of b is between 0.5 and 1.0, particularly b=0.5 means that the electrochemical reaction is a diffusion-controlled process, while b=1.0 denotes a capacitive-controlled process. The b value could be obtained through fitting the slope of log(í) vs. log(v) plot at every redox peak, which is shown in Fig. S29. Noting that the b=0.68 after fitting the slope of log(í) vs. log(v) plot, which is very close to the 0.5, indicating that the reaction is a hydrogen atom diffusion rate-controlled process.

References:

[1] J. Ye, X. Cong, P. Zhang, E. Hoffmann, G. Zeng, Y. Wu, H. Zhang and W. Fan. *Water, Air, Soil Pollut.* **2015**, *226*, 306.

[2] M. Fantauzzi, B. Elsener, D. Atzei, A. Rigoldi and A. Rossi. *RSC Adv.* **2015**, *5*, 75953.

Section S3. Supplementary Figures

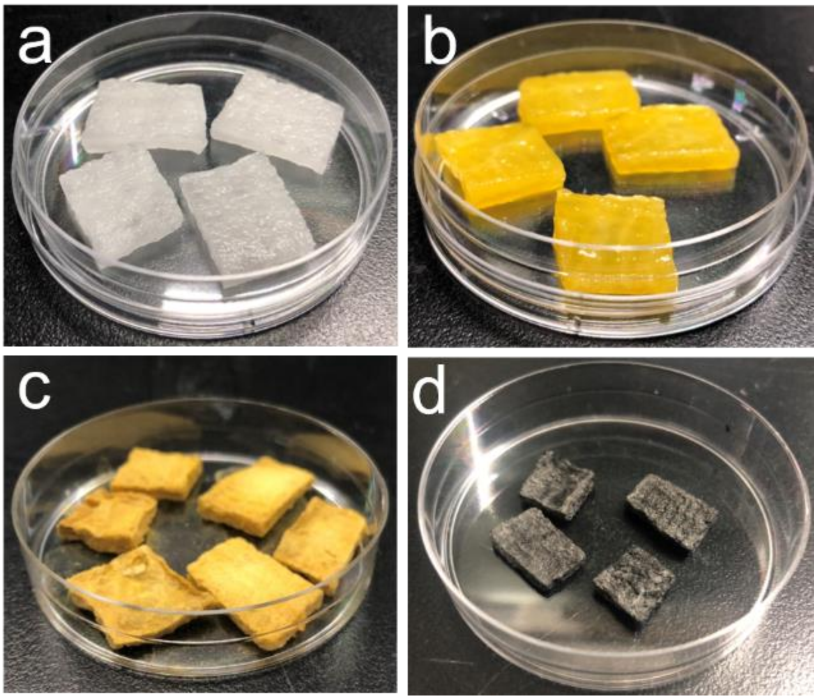

Fig. S1. Photograph of as-prepared Pt@WRC samples during the impregnated and drying progress: (a) after leached in the deionized water (b) after impregnated in the H_2PtCl_6 aqueous solution (c) the samples after freezing and drying (d) carbonized samples.

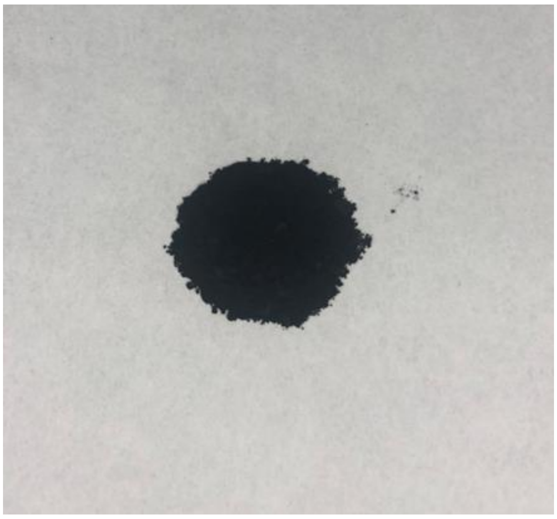

Fig. S2 Photograph of as-prepared Pt@WRC catalyst on filter paper.

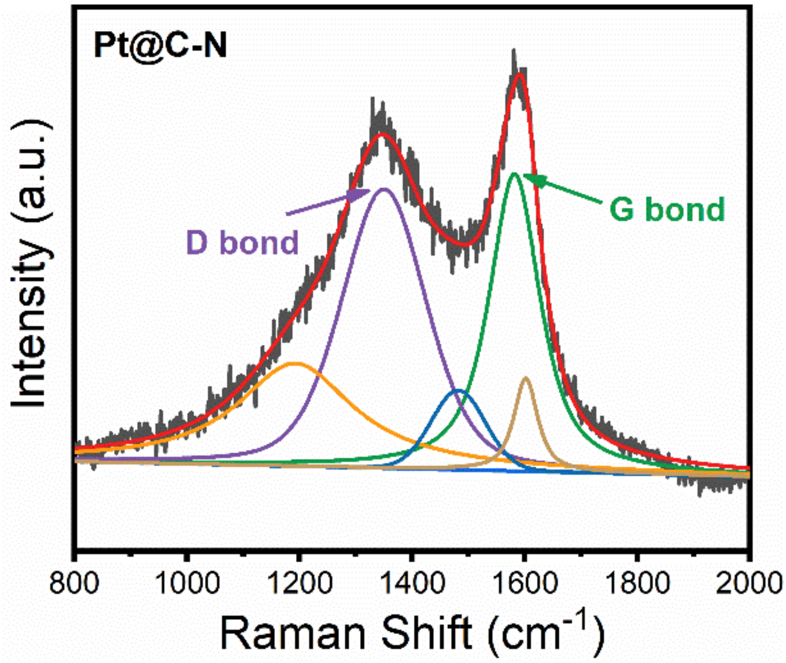

Fig. S3. Raman spectrum of the electrocatalysts of Pt@C-N-W calcined at 600 °C and impregnated in different concentration of H_2PtCl_6 aqueous solution.

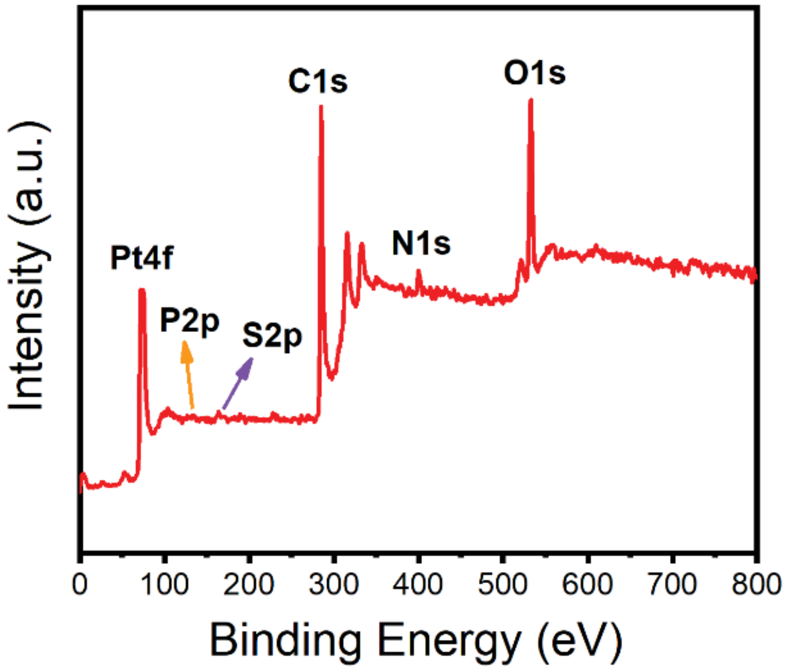

Fig. S4. The whole spectrum of the XRS of Pt@WRC calcined at 600 °C and impregnated in different concentration of H_2PtCl_6 aqueous solution.

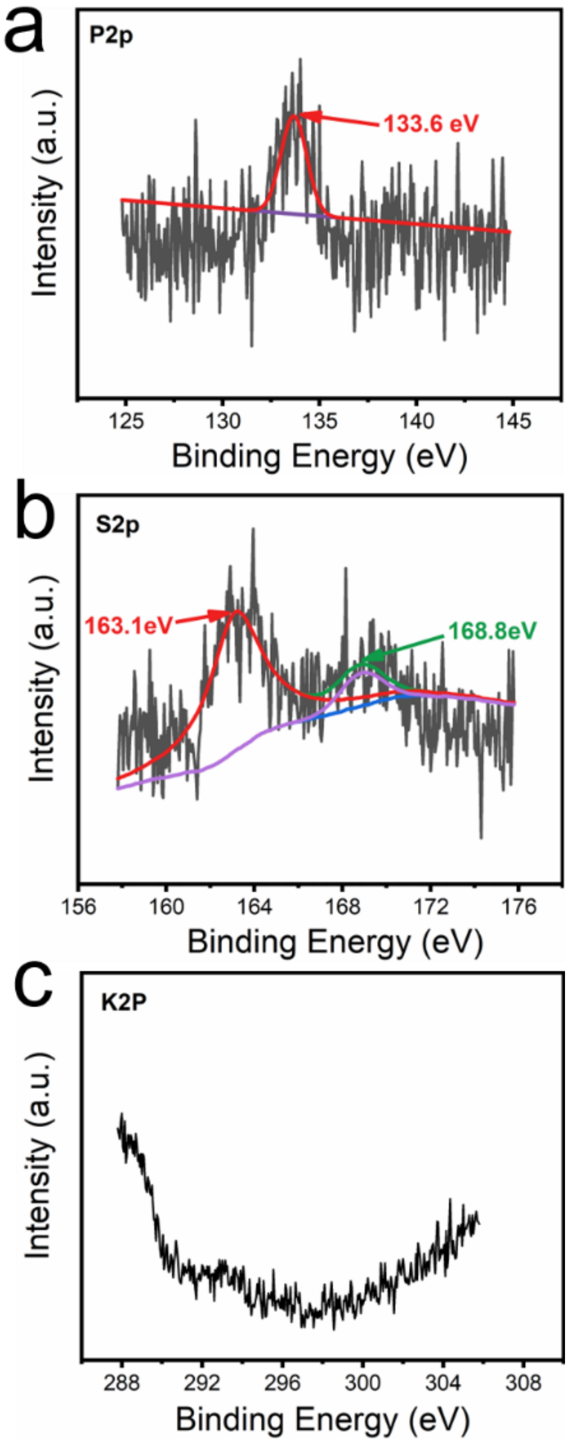

Fig. S5. XPS data of P (a), S (b), and K (c) in the Pt@C-N sample.

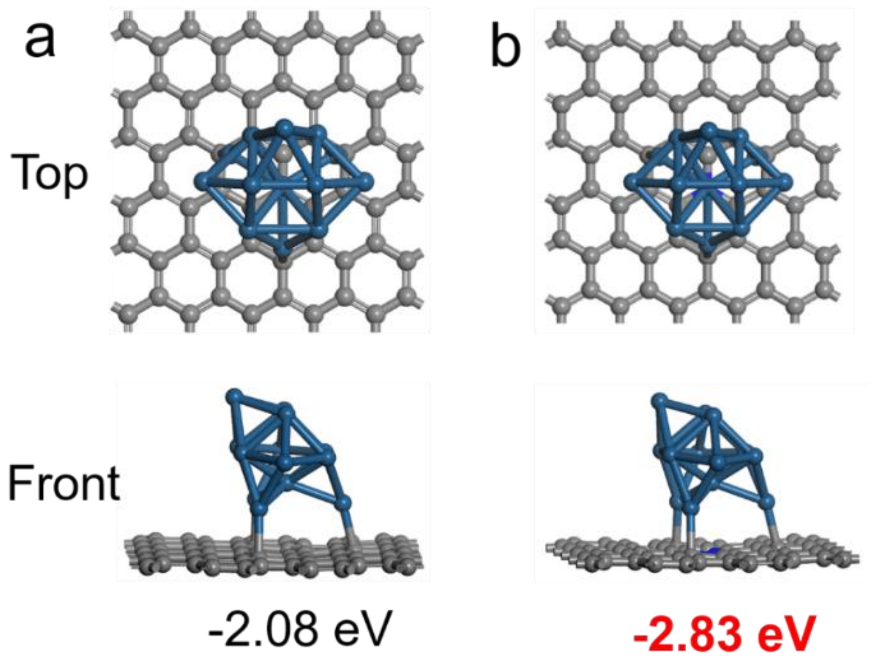

Fig. S6. The schematic diagram of the stability bonds between Pt_{13} and C (a) and C/N (b) as well as the adhesion energies during the bonding are listed in below. It demonstrates that the more energy is released when the Pt_{13} is bonded with C-N materials and presents a stable state.

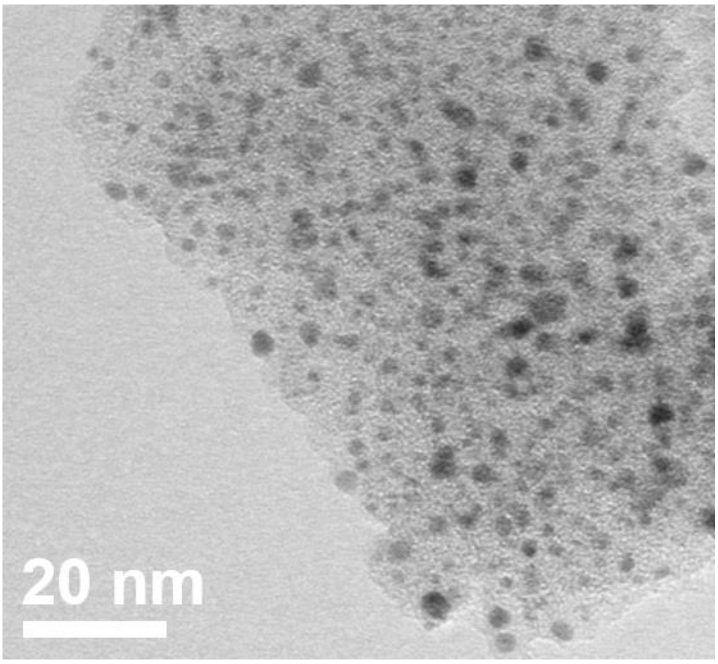

Fig. S7. TEM image of the Pt@C-N catalyst after ball milling for 24 h and sonication for 2 h.

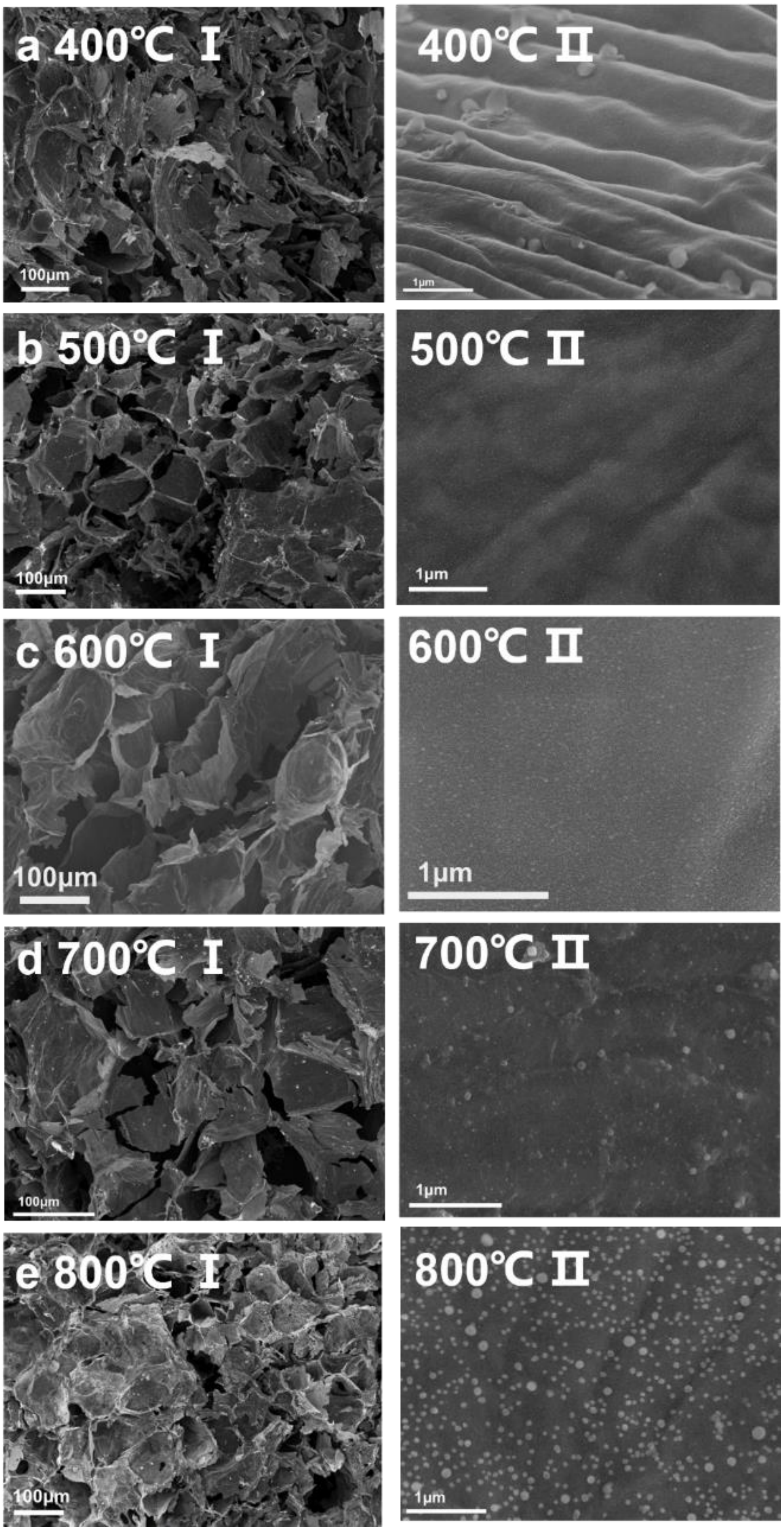

Fig. S8. SEM images of the Pt@WRC catalysts calcined at different temperature: (a) 400 °C, (b) 500 °C, (c) 600 °C, (d) 700 °C, (e) 800°C and magnification: I: ×100; II: ×20000.

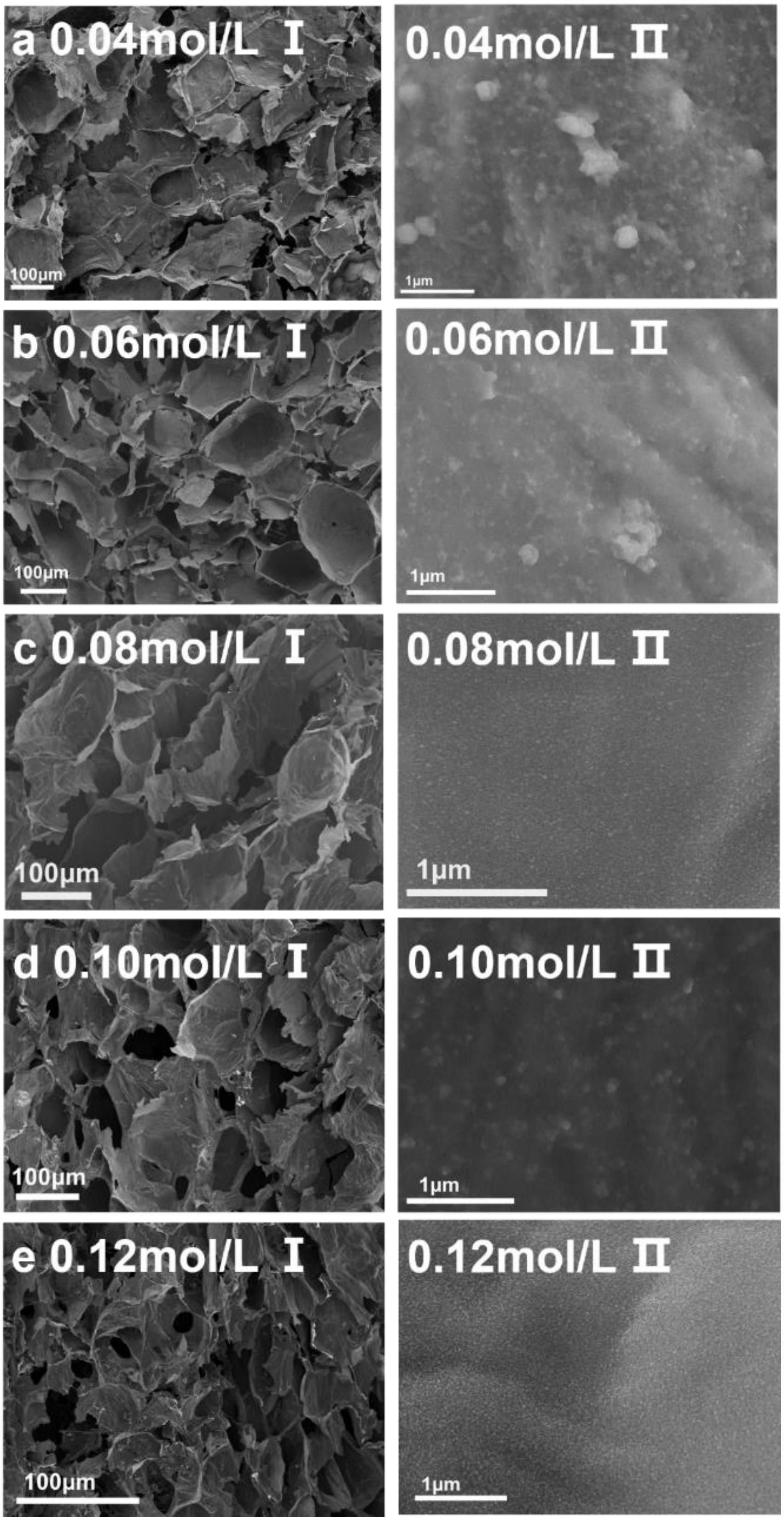

Fig. S9. SEM images of the Pt@WRC catalysts impregnated in different concentration of H_2PtCl_6 aqueous solution: (a) 0.04 mol/L, (b) 0.06 mol/L, (c) 0.08 mol/L, (d) 0.10 mol/L, (e) 0.12 mol/L and magnification: I: ×100; II: ×20000.

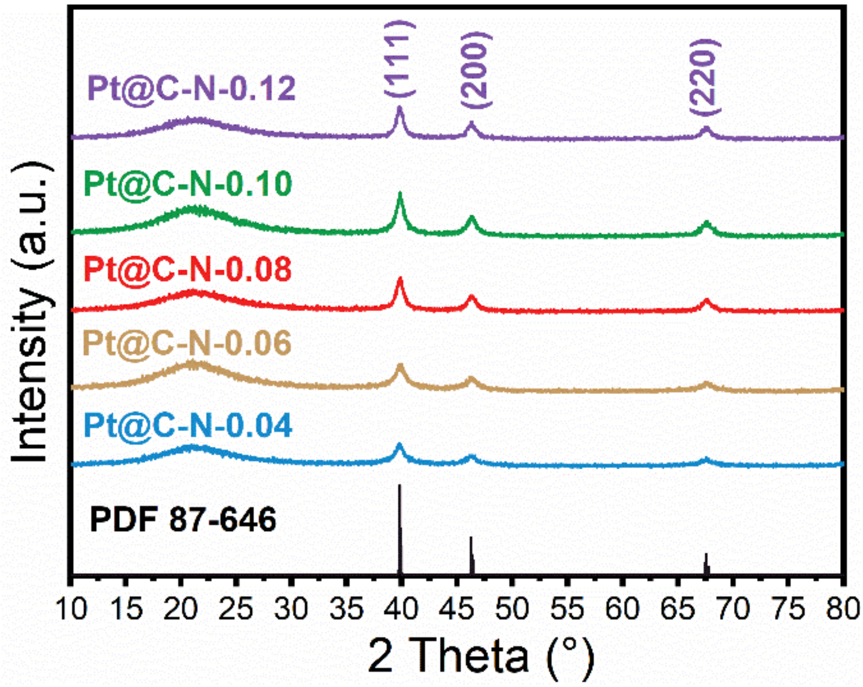

Fig. S10. XRD patterns of the electrocatalysts under different concentration of H_2PtCl_6 aqueous solution.

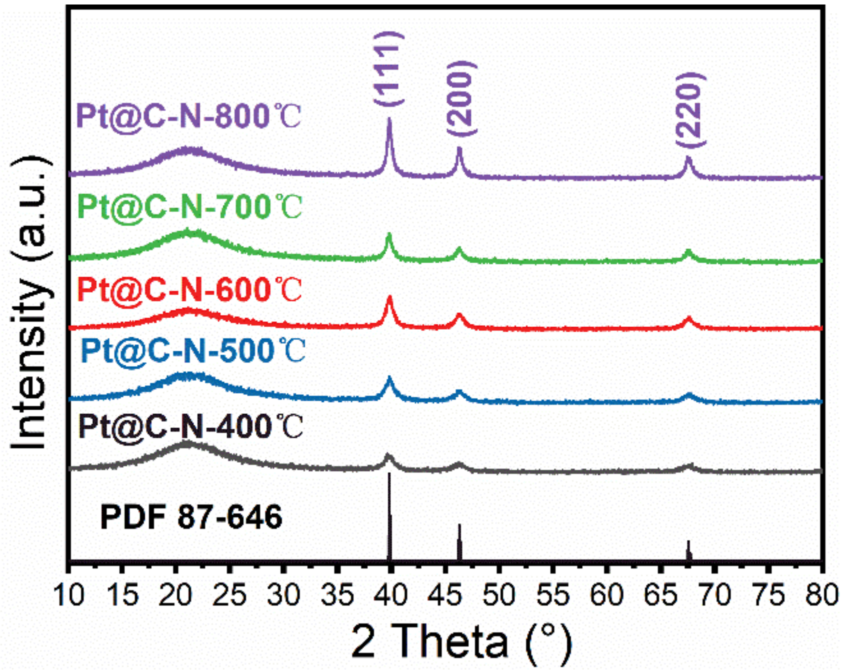

Fig. S11. XRD patterns of the electrocatalysts at different calcined temperature.

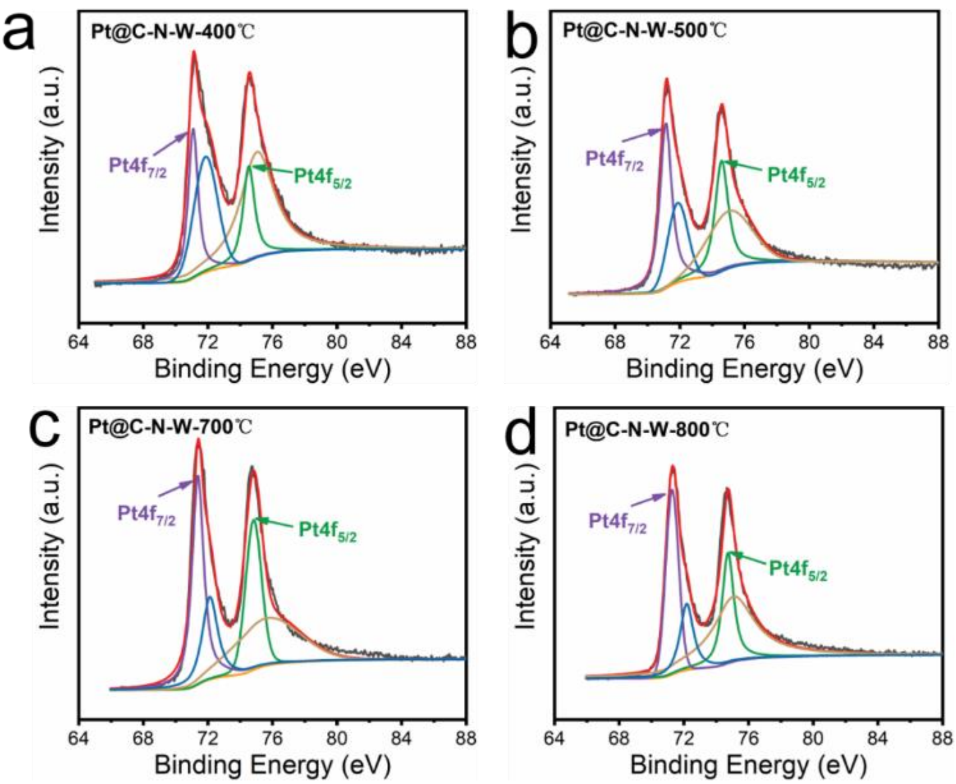

Fig. S12. XPS spectrum of the electrocatalysts calcined at different temperature (a) 400 °C (b) 500 °C (c) 700 °C (d) 800 °C.

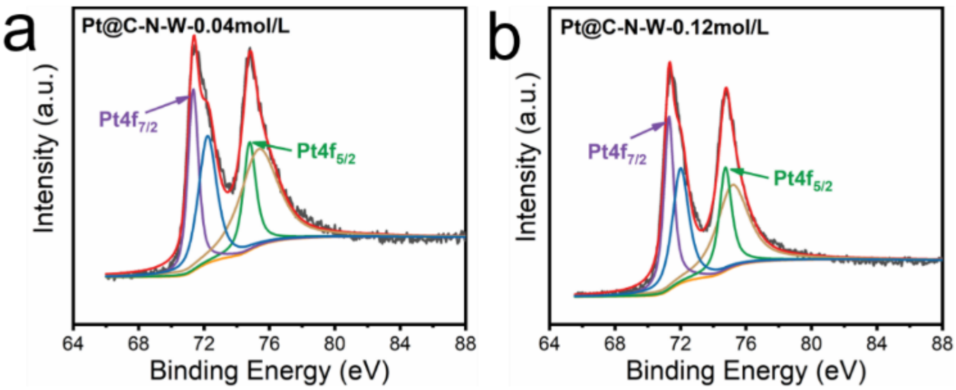

Fig. S13. XPS spectrum of the electrocatalysts calcined at impregnated in different concentration of H_2PtCl_6 aqueous solution (a) 0.04 mol/L (b) 0.12 mol/L.

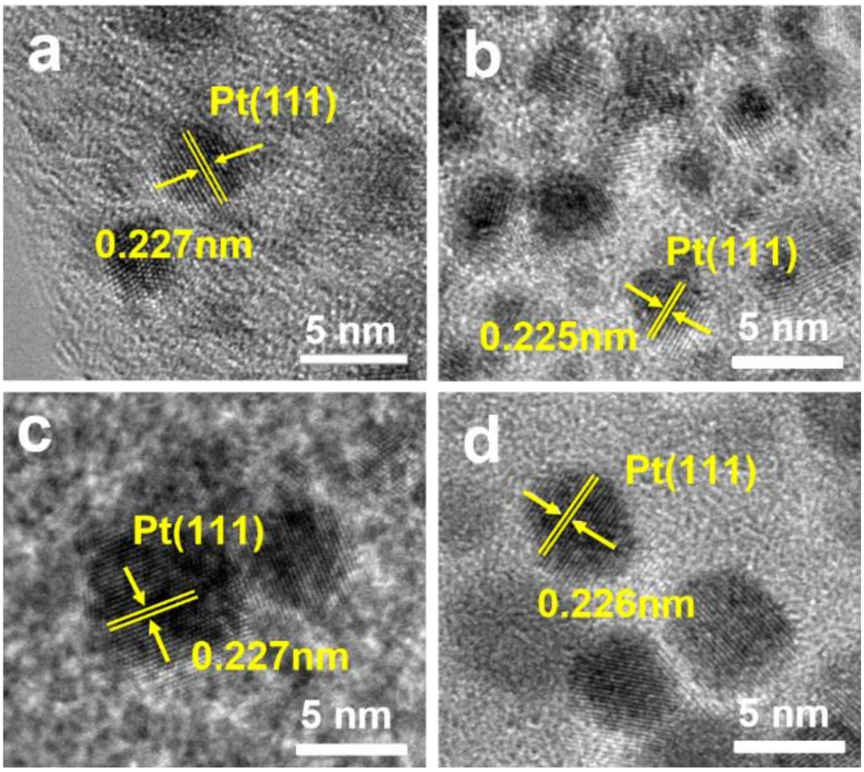

Fig. S14. HRTEM image of Pt@C-N electrocatalyst calcined at calcined at different temperature (a) 400 °C (b) 500 °C (c) 700 °C (d) 800 °C and impregnated in 0.08 mol/L H_2PtCl_6 aqueous solution.

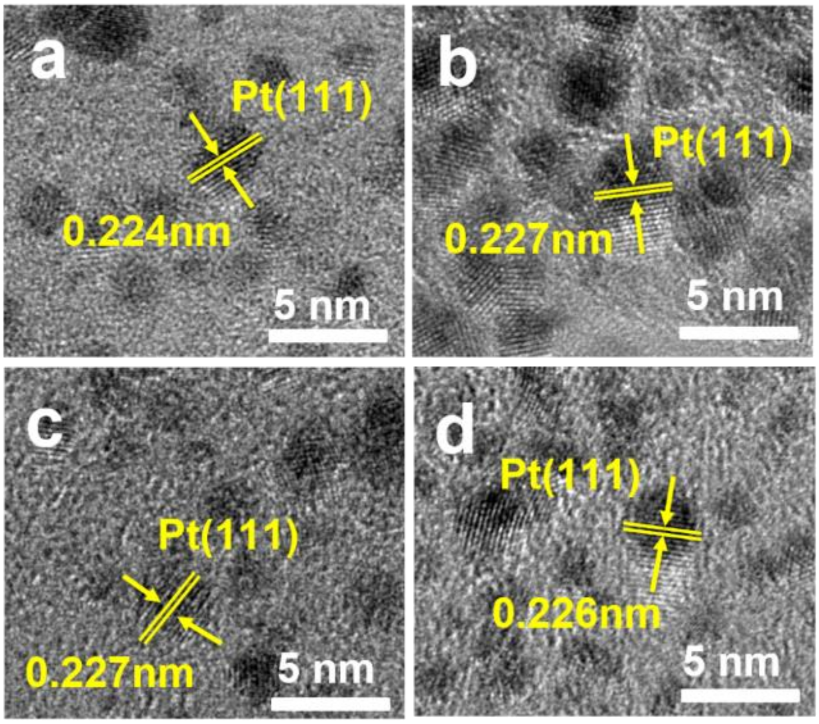

Fig. S15. HRTEM image of Pt@C-N electrocatalyst calcined at 600 °C and impregnated in different concentration of H_2PtCl_6 aqueous solution: (a) 0.04 mol/L (b) 0.06 mol/L (c) 0.10 mol/L (d) 0.12.

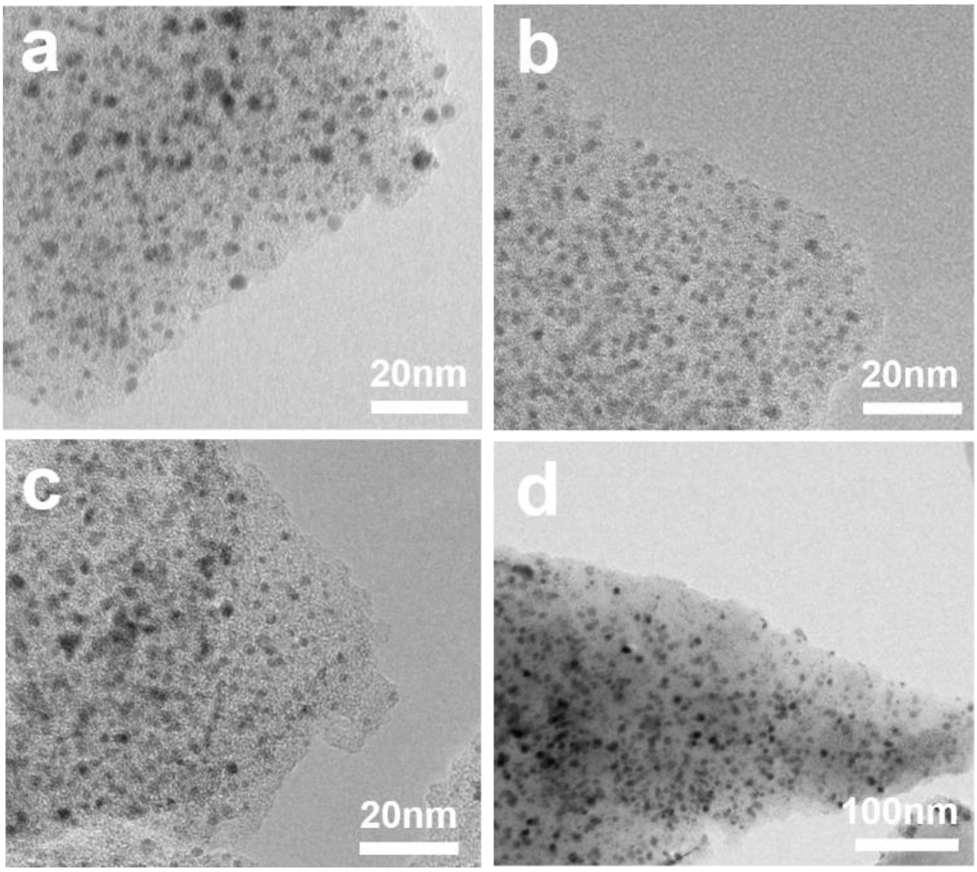

Fig. S16. TEM images of the electrocatalysts on different supported materials (a) Pt@C-N (WRC) (b) Pt@C-N (DFC) (c) Pt@C-N (SWC) (d) Pt@C-N (PTC).

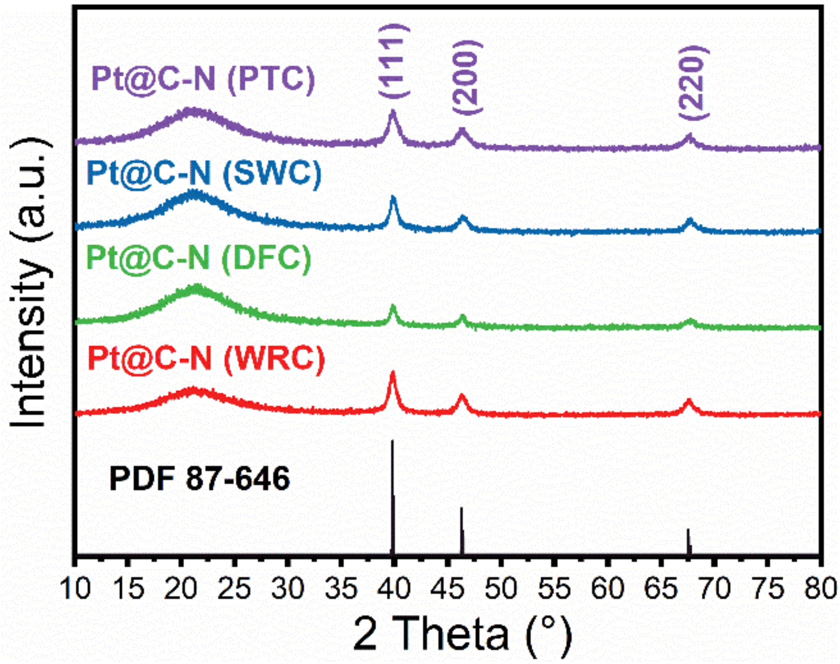

Fig. S17. XRD patterns of the electrocatalysts with different supported materials.

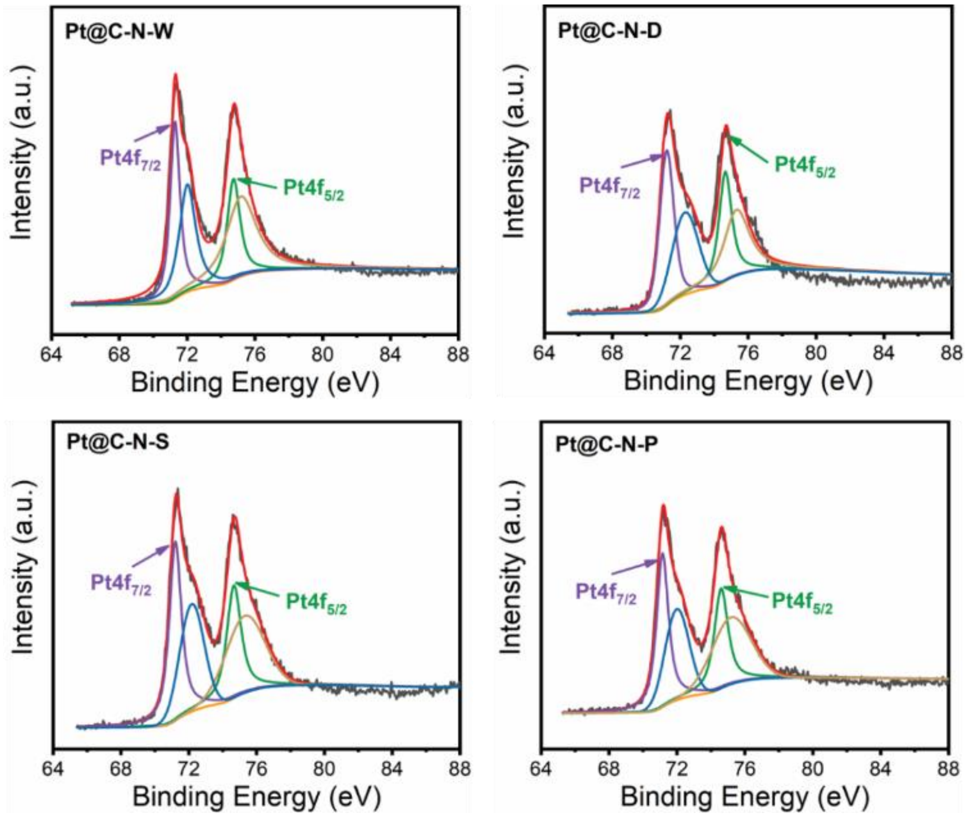

Fig. S18. XPS spectrum of the electrocatalysts on different supported materials (a) Pt@C-N-W (b) Pt@ C-N-D (c) Pt@ C-N-S (d) Pt@ C-N-P.

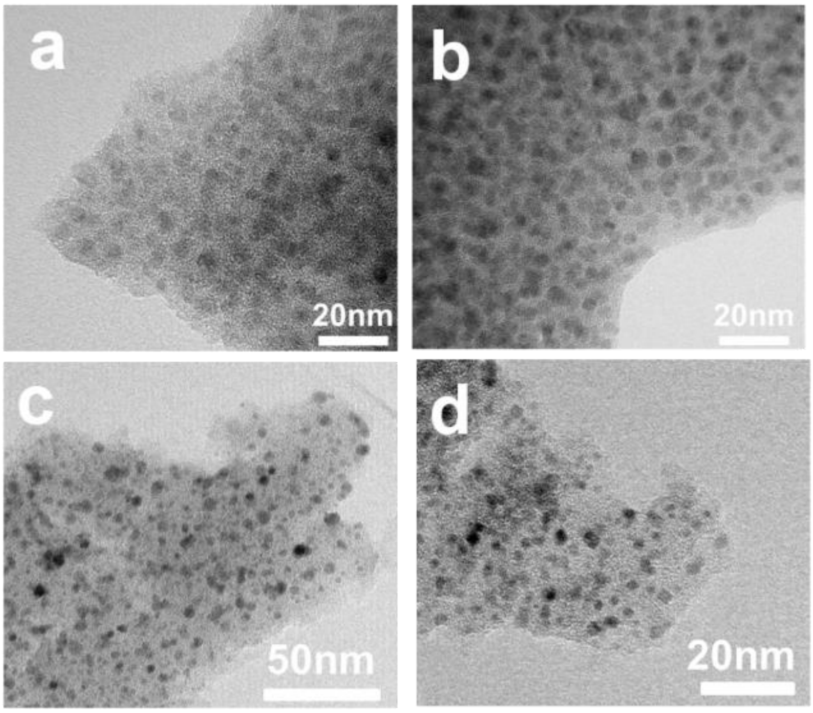

Fig. S19. TEM images of the different electrocatalysts (a) Co@C-N (b) Ni@C-N (c) Ru@C-N (d) Rh@C-N.

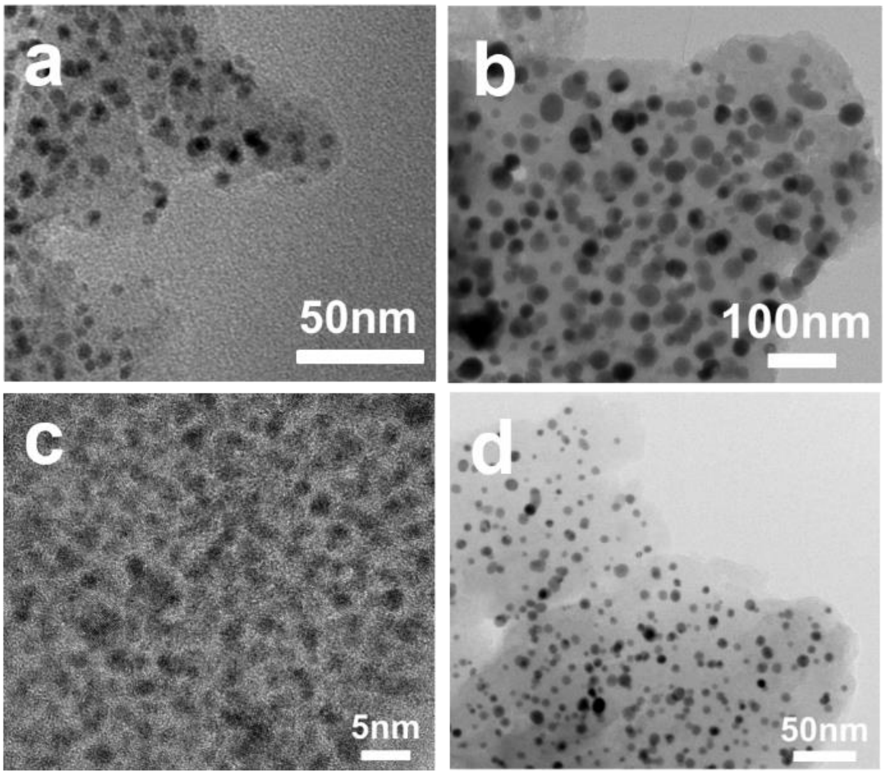

Fig. S20. TEM images of the different electrocatalysts (a) Pd@C-N (b) Ag@C-N (c) Ir@C-N (d) Au@C-N.

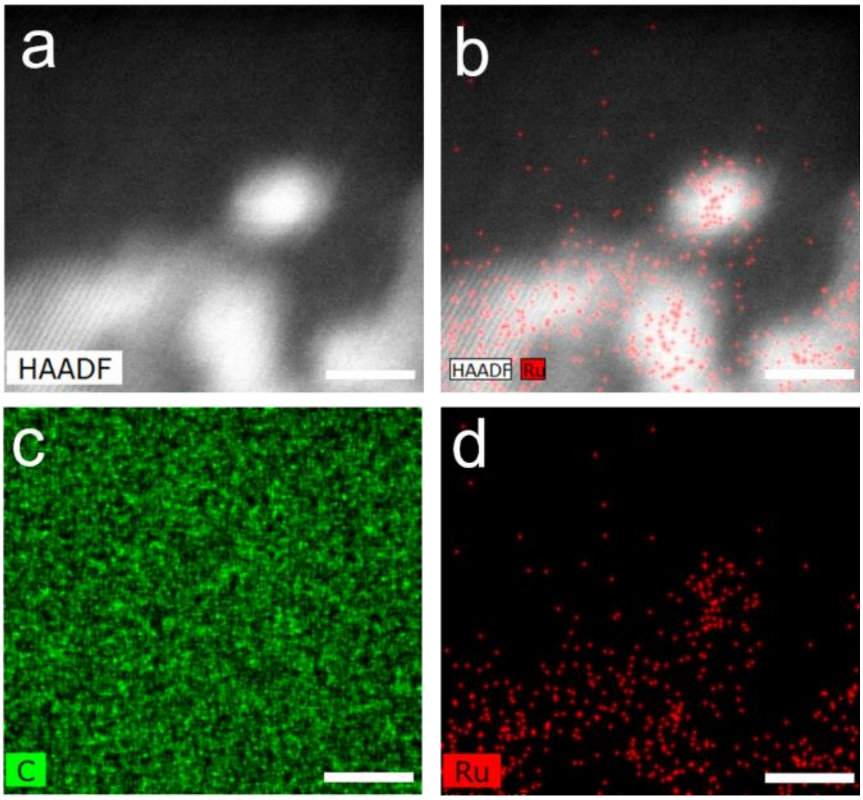

Fig. S21. STEM-EDS images of the Ru@C-N electrocatalysts, Scale bars, 3 nm.

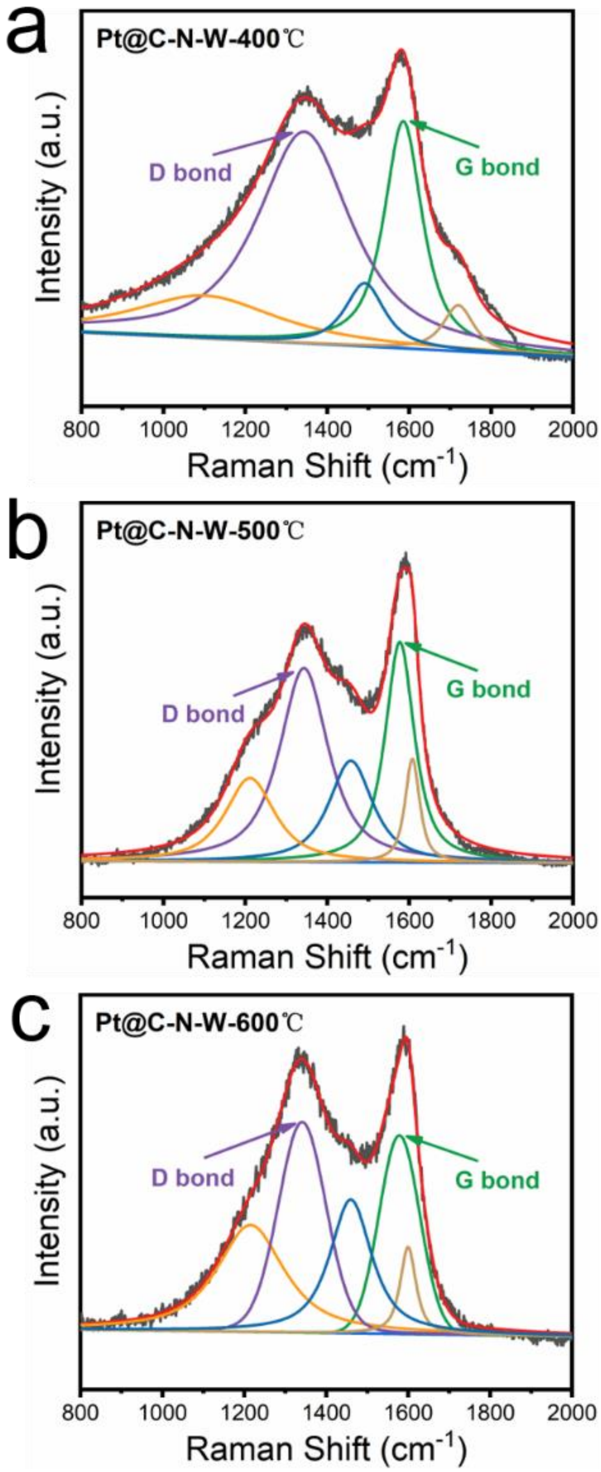

Fig. S22. Raman spectra of the Pt@C-N-W electrocatalysts calcined at different temperatures: (a) 400 ℃, (b) 500 ℃, (c) 600℃.

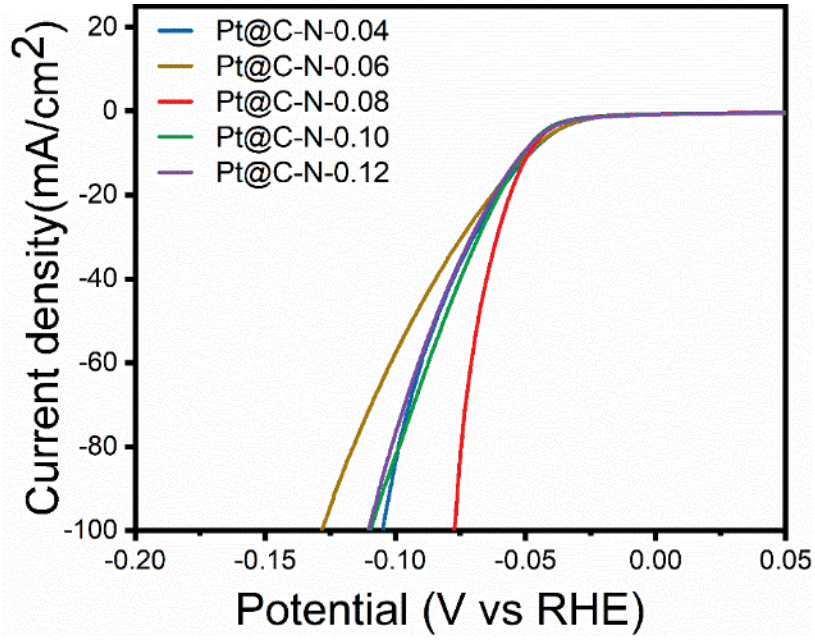

Fig. S23. Polarization curves of Pt@C-N electrocatalyst in different concentration of H_2PtCl_6 aqueous solution at the scan rate of 10 mV/s in 0.5 M H_2SO_4.

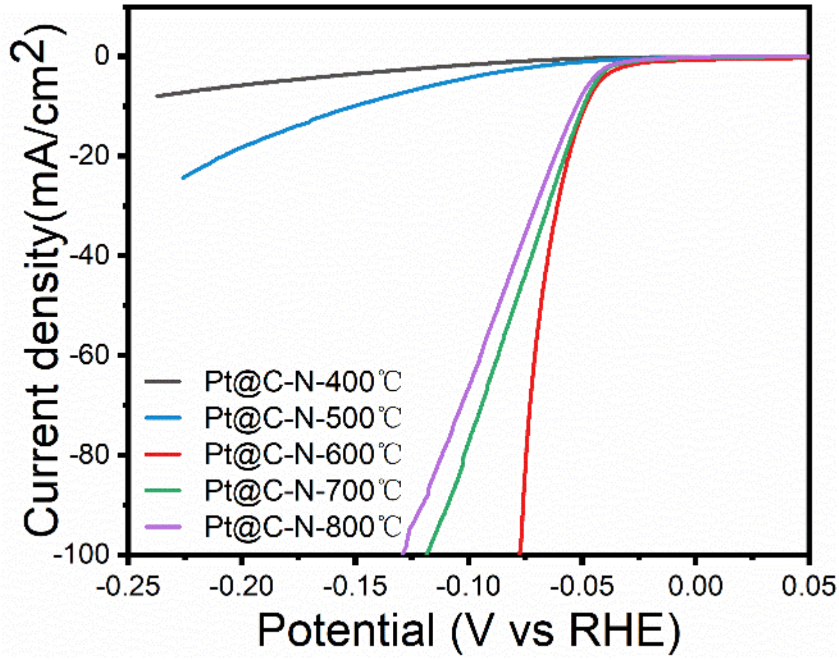

Fig. S24. Polarization curves of Pt@C-N electrocatalyst calcined at different temperature at the scan rate of 10 mV/s in 0.5 M H_2SO_4.

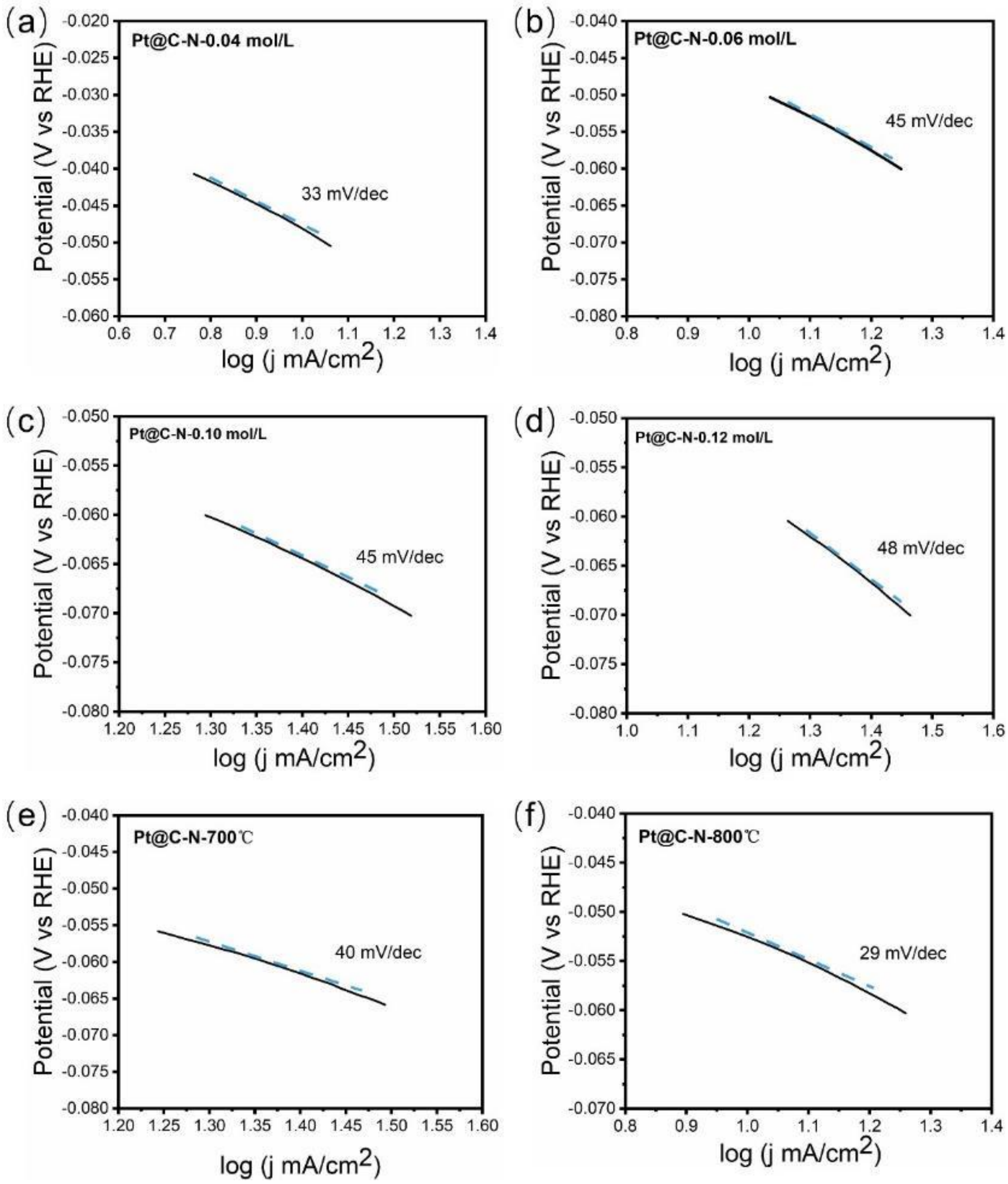

Fig. S25. Tafel slop of Pt@C-N electrocatalysts under different concentration of H_2PtCl_6 aqueous solution (a) 0.04 mol/L (b) 0.06 mol/L (c) 0.10mol/L (d) 0.12 mol/L and calcined at different temperature (e) 700 °C (f) 800 °C.

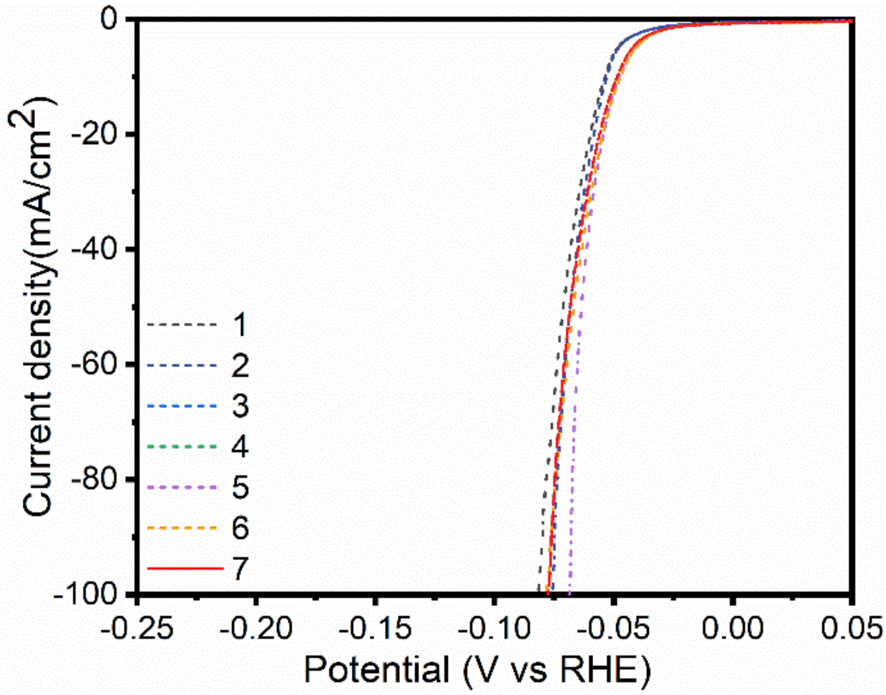

Fig. S26. HER activity of different Pt@C-N samples with same Pt mass loading. The performance of the Pt@C-N electrocatalyst is reproducible.

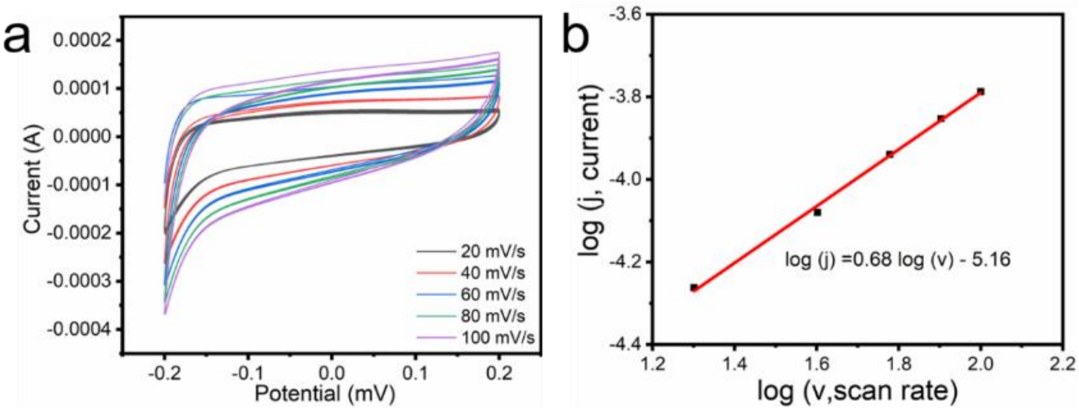

Fig. S27. CV curves of Pt@C-N catalyst at different scanning rate (a) and log (i) versus log (v) of the as-prepared Pt@C-N (b); the slope of the fitting line is 0.68, which is close to the 0.5, indicating the reaction is a hydrogen atom diffusion rate-controlled process.

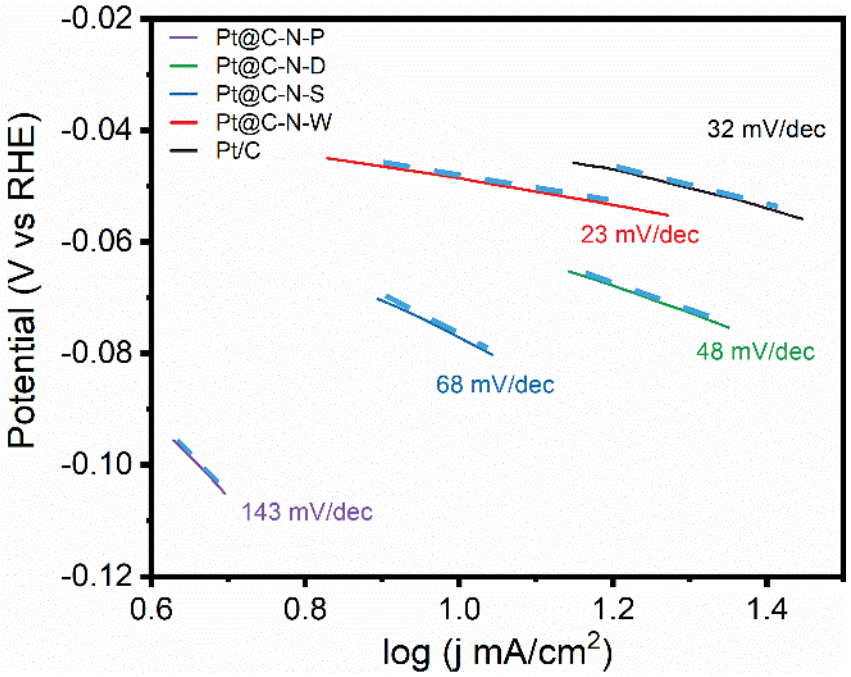

Fig. S28. Tafel slopes curves of electrocatalysts on various matrix materials calcined at 600 °C and impregnated in 0.08 mol/L H_2PtCl_6 aqueous solution at the scan rate of 10 mV/s in 0.5 M H_2SO_4.

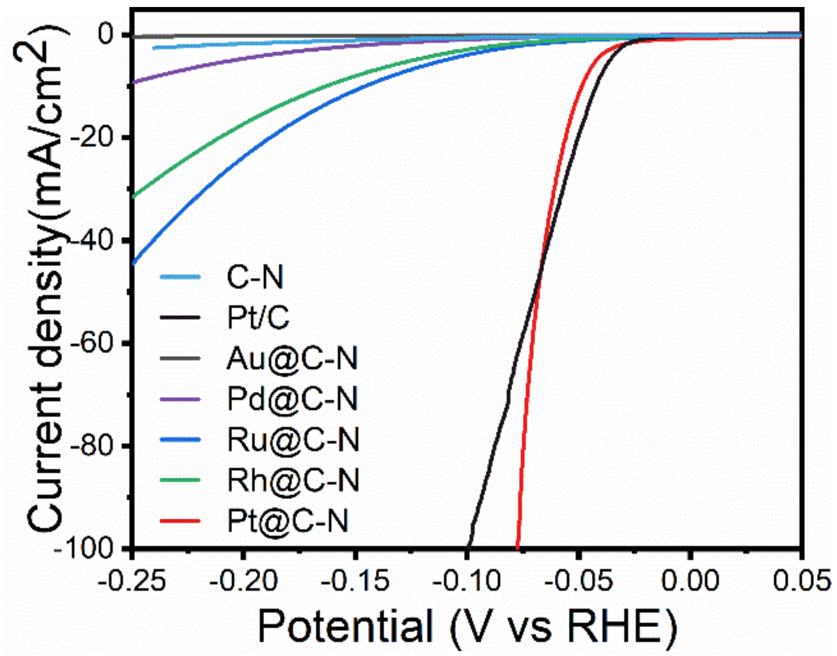

Fig. S29. Polarization curves of diverse metal-based electrocatalysts calcined at 600 °C and impregnated in 0.08 mol/L different metal aqueous solution at the scan rate of 10 mV/s in 0.5 M H_2SO_4.

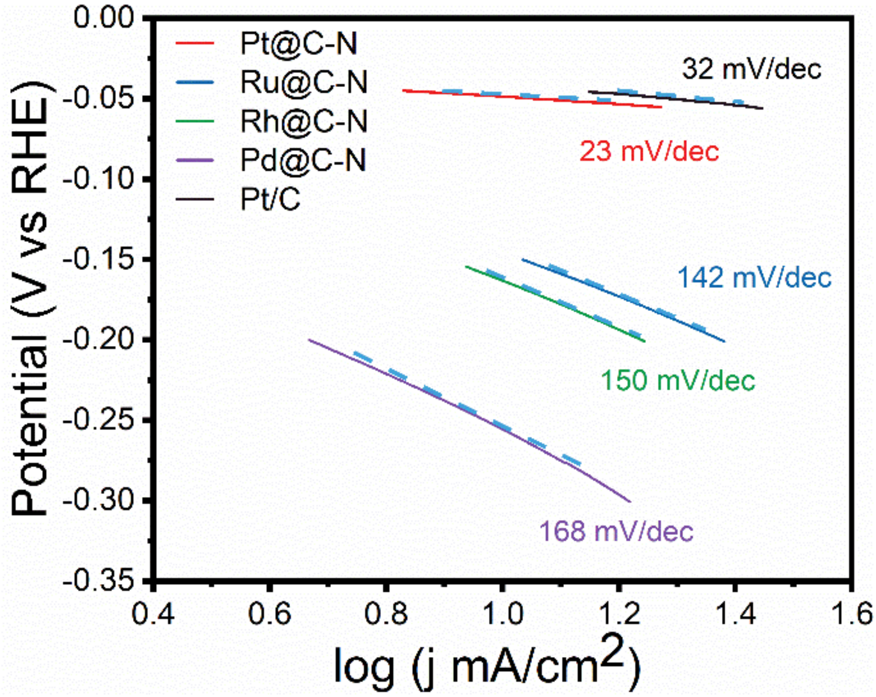

Fig. S30. Tafel slopes curves of diverse metal-based electrocatalysts calcined at 600 °C and impregnated in 0.08 mol/L different metal aqueous solution at the scan rate of 10 mV/s in 0.5 M H_2SO_4.

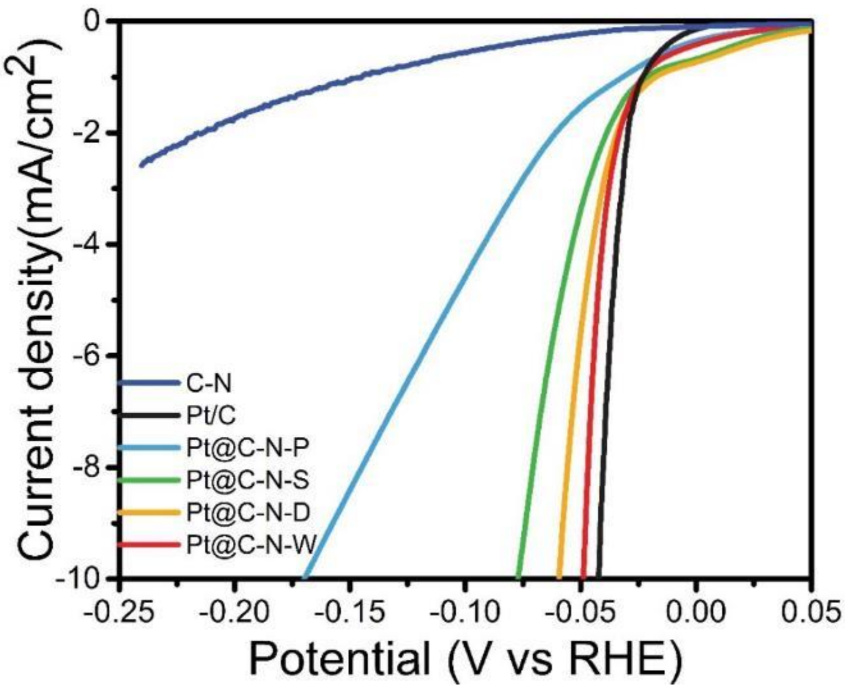

Fig. S31. Local enlarged polarization curves of electrocatalysts on various matrix materials calcined at 600 °C and impregnated in 0.08 mol/L H_2PtCl_6 aqueous solution at the scan rate of 10 mV/s in 0.5 M H_2SO_4 at the current density of 10 mA/cm^2.

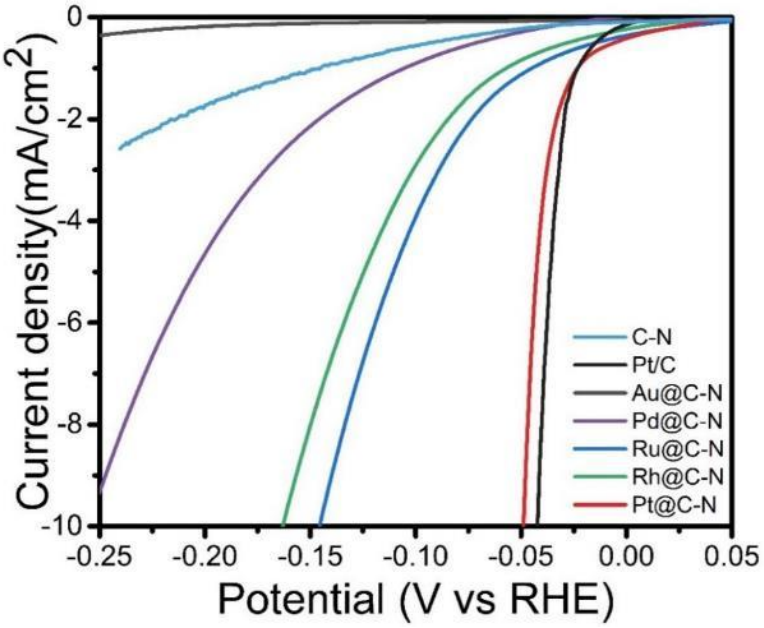

Fig. S32. Local enlarged polarization curves of the diverse metal-based electrocatalysts calcined at 600 °C and impregnated in 0.08 mol/L different metal aqueous solution at the scan rate of 10 mV/s in 0.5 M H_2SO_4 at the current density of 10 mA/cm^2.

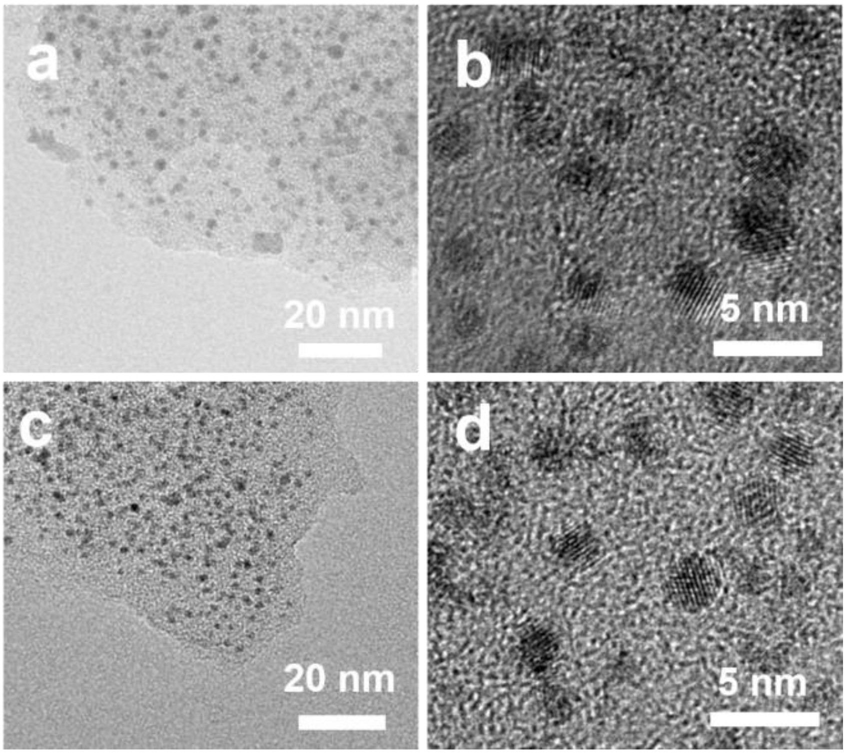

Fig. S33. TEM images of Pt@C-N before (a)-(b) and after (c)-(d) stability test. The Pt nanoparticles in Pt@C-N could still distribute uniformly on the carbon support, and no obvious agglomerate could be investigated after the long-term electrochemical testing.

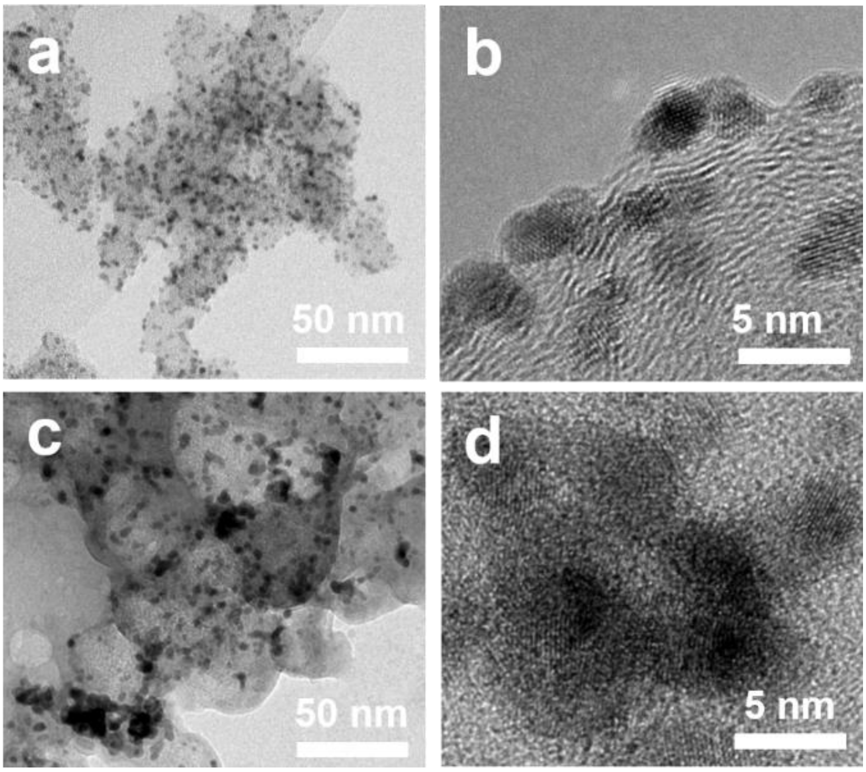

Fig. S34. TEM images of commercial Pt/C before (a)-(b) and after (c)-(d) stability test. The Pt nanoparticles in commercial Pt/C agglomerated after the long-term testing, which indicates that agglomeration of Pt nanoparticles might lead to the decreasing of the deactivation in catalyst.

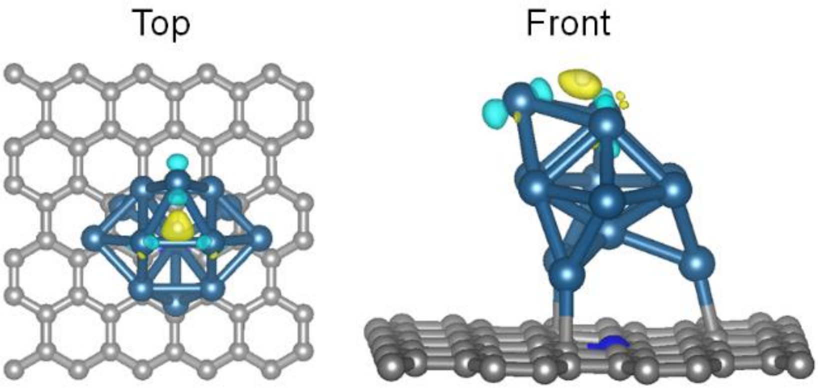

Fig. S35. Charge density difference maps for H adsorption onto the right above of the Pt_{13} (111) plate. The yellow region represents charge accumulation and the cyan region represents charge depletion. It demonstrates that charge transfer from Pt to H atom.

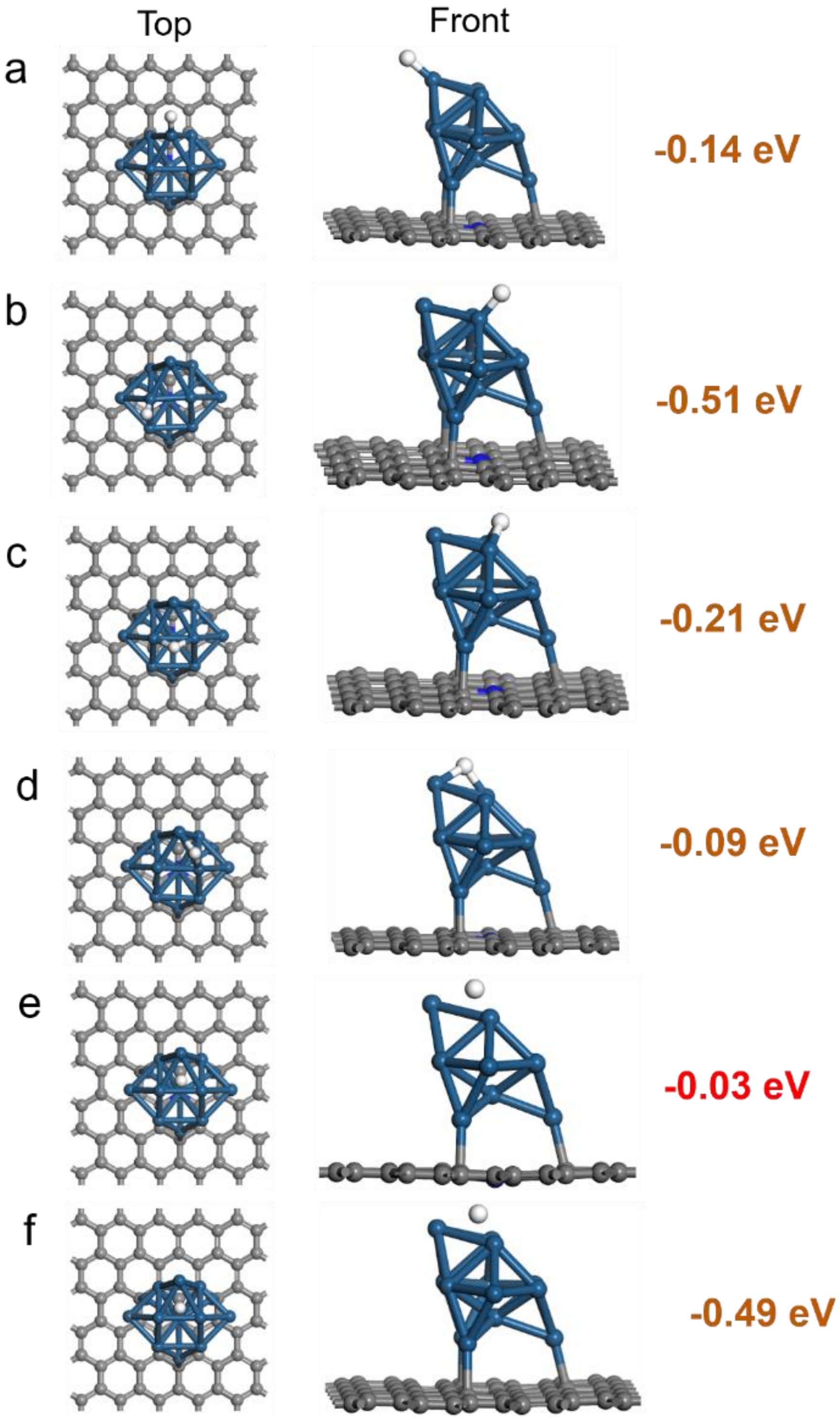

Fig. S36. The schematic diagram of the H atom is absorbed at different sites of Pt_{13} (111) plate: (a) the highest Pt_{13} atom (b) another Pt_{13} atom expect the highest (c) above the middle bridge between two atoms (d) another middle bridge between two atoms (e) the right above of the Pt_{13} (111) plate, (f) the right above of the Pt_{13} (111) plate of pure C and the absorption energies are listed below each diagram. The lowest absorption energy is -0.03 eV when the H atom is adsorbed at the right above of the Pt_{13} (111) plate.

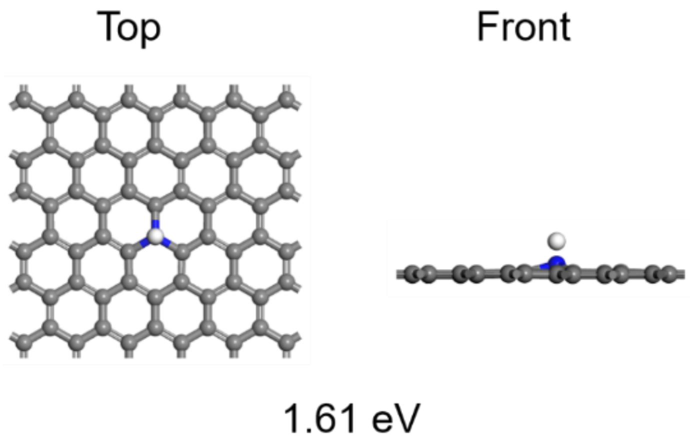

Fig. S37. Structures of H atom adsorbed on the C/N materials.

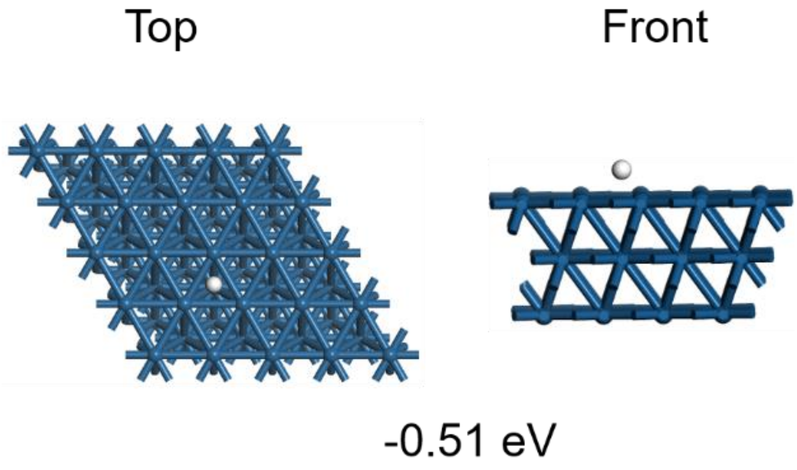

Fig. S38. Structures of H atom adsorbed on the pure Pt_{13}.

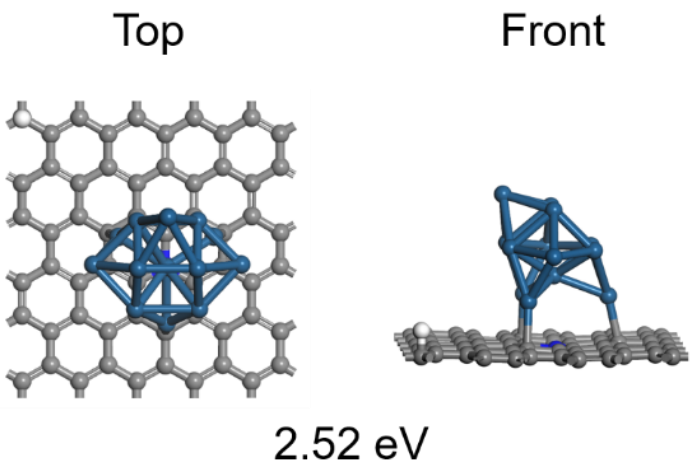

Fig. S39. Structures of H atom adsorbed on the C of Pt@C-N catalyst.

Section S4. Supplementary Equations

Eq. S1:

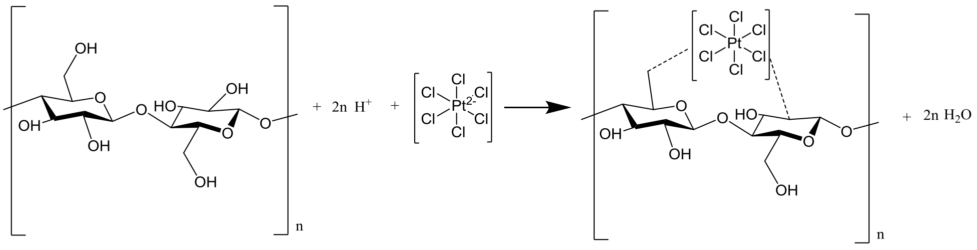

Eq. S2:

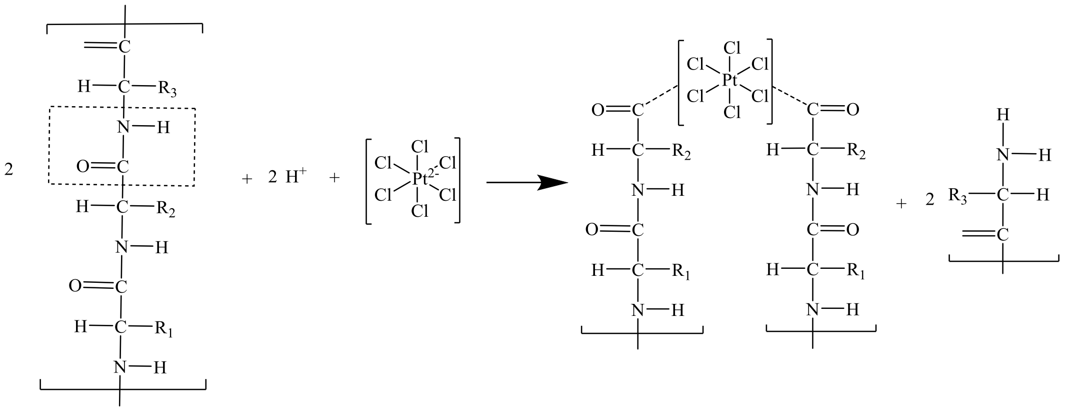

Eq. S3:

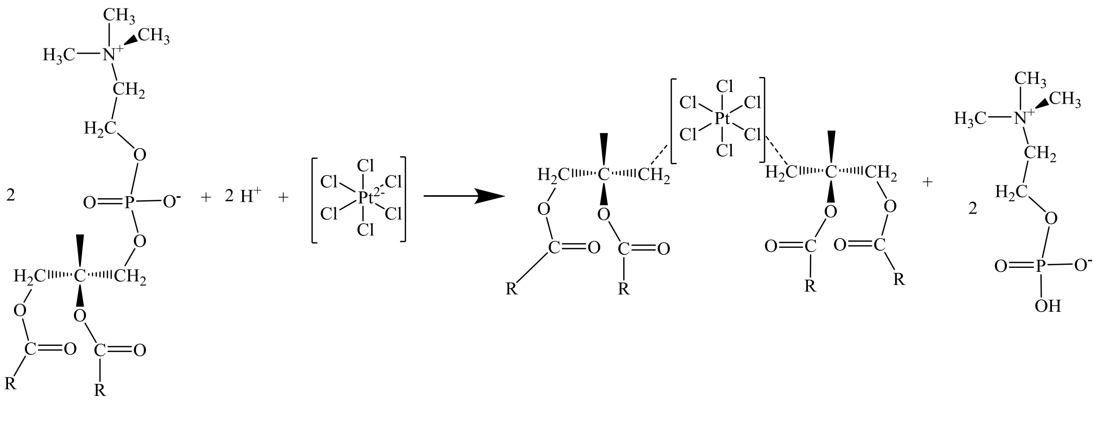

Section S5. Supplementary Tables

Table S1: The $R_{ID/IG}$ values calculated from the Raman spectra of the Pt@C-N-W electrocatalysts calcined at different temperatures.

Heating temperature (℃)	$R_{ID/IG}$
400	2.28
500	1.47
600	1.32

The results indicate that the degree of graphitization of the carbon support increases with the increasing calcined temperatures, which might contribute a better electron transfer for the electrochemical reaction and finally result in a higher catalytic activity for the samples calcined at 600 ℃.

Table S2: Summary of some recently reported representative HER electrocatalysts in 0.5 M H_2SO_4 electrolytes.

Catalysts	Catalysts loading (wt%)	Catalysts loading on GC (mg/cm^2)	Overpotential at 10 mA cm^{-2} (mV)	Overpotential at 100 mA cm^{-2} (mV)	Tafel slope (mV/dec)	Ref.
Pt@C-N	22.8	0.058	49	77	23	This work
Pt/C	20	0.051	42	100	32	This work
Pt-MoS_2	36	0.075	53	-	40	1
Pt MLAg NF/Ni foam	-	-	70	-	53	2
PtNPS/CNFs	7		175	-	50	3
Pt_1@MC	2.6	0.01	25	65	26	4
A-Ni@DG	1.24	0.262	70	-	31	5
Co_1/PCN	0.3	0.5	151	-	52	6
CoPS	-	-	48	-	57	7
$MoS_2/CoSe_2$	-	0.28	68	-	36	8
α-INS	-	0.254	105	-	40	9
Mo_2C@NPC/NPRGO	-	0.14	34	-	33.6	10
MoC_x	-	0.8	142	236	53	11
P-WN/rGO	-	0.337	85	260	54	12
SV-MoS_2	-	-	170	-	60	13
Ru-HPC	5.55	0.4	61.6	170	66.8	14
CoS_2 NW	-	-	145	215	51.6	15
Ru@C_2N	28.7	0.285	22	-	30	16
RhCo	18	-	28.1	-	24	17
SCEIN/SWNT	5.6	0.18	77	-	40	18
Co-NG	-	0.285	147	-	82	19
Pt1/OLC	0.27	0.51	38	-	36	20
Pt-TiS_2	-	-	75	-	40.6	21
VA-MoS_2@Pt	-	-	91	200	24	22
Pt_1@Fe-N-C	2.1	3	60	-	42	23
NS-doped nanoporous graphene	-	-	390	-	105	24
P-MoS_2	-	0.213	219	-	39	25
np-Ni/graphene	-	-	50	-	45	26
ALDPt/NGNs	2.1	-	50	-	29	27
$Fe_{1-x}Co_xS_2$/CNT	-	0.4	120	170	46	28
Ni_2P	-	1.0	130	180	30	29
Ru@GnP	18.7	0.75	13	-	30	30

References

[1] X. Huang, Z. Zeng, S. Bao, M. Wang, X. Qi, Z. Fan and H. Zhang. *Nat. Commun.* **2013**, *4*, 1444.

[2] M. Li, Q. Ma, W. Zi, X. Liu, X. Zhu and S. F. Liu. *Sci. Adv.* **2015**, *1*, 8.

[3] T. Yang, M. Du, H. Zhu, M. Zhang and M. Zou. *Electrochim. Acta* **2015**, *167*, 48.

[4] H. Wei, K. Huang, D. Wang, R. Zhang, B. Ge, J. Ma, B. Wen, S. Zhang, Q. Li, M. Lei, C. Zhang, J. Irawan, L. M. Liu and H. Wu. *Nat. Commun.* **2017**, *8*, 1490.

[5] L. Zhang, Y. Jia, G. Gao, X. Yan, N. Chen, J. Chen, M. T. Soo, B. Wood, D. Yang, A. Du and X. Yao. *Chem.* **2018**, 4, 285.

[6] L. Cao, Q. Luo, W. Liu, Y. Lin, X. Liu, Y. Cao, W. Zhang, Y. Wu, J. Yang, T. Yao and S. Wei. *Nat. Catal.* **2018**, 2, 134.

[7] M. Caban-Acevedo, M. L. Stone, J. R. Schmidt, J. G. Thomas, Q. Ding, H. C. Chang, M. L. Tsai, J. H. He and S. Jin. *Nat. Mater.* **2015**, 14, 1245.

[8] M. R. Gao, J. X. Liang, Y. R. Zheng, Y. F. Xu, J. Jiang, Q. Gao, J. Li and S. H. Yu. *Nat. Commun.* **2015**, 6, 5982.

[9] X. Long, G. Li, Z. Wang, H. Zhu, T. Zhang, S. Xiao, W. Guo and S. Yang. *J. Am. Chem. Soc.* **2015**, *137*, 11900.

[10] J.-S. Li, Y. Wang, C.-H. Liu, S.-L. Li, Y.-G. Wang, L.-Z. Dong, Z.-H. Dai, Y.-F. Li and Y.-Q. Lan. *Nat. Commun.* **2016**, 7, 11204.

[11] H. B. Wu, B. Y. Xia, L. Yu, X.-Y. Yu and X. W. Lou. *Nat. Commun.* **2015**, 6, 6512.

[12] H. Yan, C. Tian, L. Wang, A. Wu, M. Meng, L. Zhao and H. Fu. *Angew Chem. Int. Ed. Engl.* **2015**, *54*, 6325.

[13] H. Li, C. Tsai, A. L. Koh, L. Cai, A. W. Contryman, A. H. Fragapane, J. Zhao, H. S. Han, H. C. Manoharan, F. Abild-Pedersen, J. K. Norskov and X. Zheng. *Nat. Mater.* **2016**, 15, 48.

[14] T. Qiu, Z. Liang, W. Guo, S. Gao, C. Qu, H. Tabassum, H. Zhang, B. Zhu, R. Zou and Y. Shao-Horn. *Nano Energy* **2019**, 58, 1.

[15] M. S. Faber, R. Dziedzic, M. A. Lukowski, N. S. Kaiser, Q. Ding and S. Jin. *J. Am. Chem. Soc.* **2014**, 136, 10053.

[16] J. Mahmood, F. Li, S. M. Jung, M. S. Okyay, I. Ahmad, S. J. Kim, N. Park, H. Y. Jeong and J. B. Baek. *Nat. Nanotechnol.* **2017**, 12, 441.

[17] J. Du, X. Wang, C. Li, X.-Y. Liu, L. Gu and H.-P. Liang. *Electrochim. Acta* **2018**, 282, 853.

[18] M. Tavakkoli, T. Kallio, O. Reynaud, A. G. Nasibulin, C. Johans, J. Sainio, H. Jiang, E. I. Kauppinen and K. Laasonen. *Angew Chem. Int. Ed. Engl.* **2015**, *54*, 4535.

[19] H. Fei, J. Dong, M. J. Arellano-Jimenez, G. Ye, N. Dong Kim, E. L. Samuel, Z. Peng, Z. Zhu, F. Qin, J. Bao, M. J. Yacaman, P. M. Ajayan, D. Chen and J. M. Tour. *Nat. Commun.* **2015**, 6, 8668.

[20] D. Liu, X. Li, S. Chen, H. Yan, C. Wang, C. Wu, Y. A. Haleem, S. Duan, J. Lu, B. Ge, P. M. Ajayan, Y. Luo, J. Jiang and L. Song. *Nat. Energy* **2019**, 4, 512.

[21] Z. Zeng, C. Tan, X. Huang, S. Bao and H. Zhang. *Energy Environ. Sci.* **2014**, *7*, 797.

[22] V. Shokhen and D. Zitoun. *Electrochim. Acta* **2017**, 257, 49.

[23] X. Zeng, J. Shui, X. Liu, Q. Liu, Y. Li, J. Shang, L. Zheng and R. Yu. *Adv. Energy Mater.* **2018**, 8, 1701345.

[24] Y. Ito, W. Cong, T. Fujita, Z. Tang and M. Chen. *Angew Chem. Int. Ed. Engl.* **2015**, 54, 2131.

[25] D. Wang, Y. Xie and Z. Wu. *Nanotechnology* **2019**, *30*, 205401.

[26] H. J. Qiu, Y. Ito, W. Cong, Y. Tan, P. Liu, A. Hirata, T. Fujita, Z. Tang and M. Chen. *Angew Chem. Int. Ed. Engl.* **2015**, *54,* 14031.

[27] N. Cheng, S. Stambula, D. Wang, M. N. Banis, J. Liu, A. Riese, B. Xiao, R. Li, T. K. Sham, L. M. Liu, G. A. Botton and X. Sun. *Nat. Commun.* **2016**, 13638.

[28] D. Y. Wang, M. Gong, H. L. Chou, C. J. Pan, H. A. Chen, Y. Wu, M. C. Lin, M. Guan, J. Yang, C. W. Chen, Y. L. Wang, B. J. Hwang, C. C. Chen and H. Dai. *J. Am. Chem. Soc.* **2015**, *137*, 1587.

[29] E. J. Popczun, J. R. McKone, C. G. Read, A. J. Biacchi, A. M. Wiltrout, N. S. Lewis and R. E. Schaak. *J. Am. Chem. Soc.* **2013**, *135*, 9267.

[30] F. Li, G. F. Han, H. J. Noh, I. Ahmad, I. Y. Jeon and J. B. Baek. *Adv. Mater.* **2018** , e1803676.
